# The chemokine CX3CL1 promotes trafficking of dendritic cells through inflamed lymphatics

**DOI:** 10.1242/jcs.135343

**Published:** 2013-11-15

**Authors:** Louise A. Johnson, David G. Jackson

**Affiliations:** Medical Research Council Human Immunology Unit, Weatherall Institute of Molecular Medicine, John Radcliffe Hospital, Headington, Oxford OX3 9DS, UK

**Keywords:** Lymphatic, Chemokine, DC trafficking, Inflammation, Endothelial cell

## Abstract

Tissue inflammation is characterised by increased trafficking of antigen-loaded dendritic cells (DCs) from the periphery via afferent lymphatics to draining lymph nodes, with a resulting stimulation of ongoing immune responses. Transmigration across lymphatic endothelium constitutes the first step in this process and is known to involve the chemokine CCL21 and its receptor CCR7. However, the precise details of DC transit remain obscure and it is likely that additional chemokine-receptor pairs have roles in lymphatic vessel entry. Here, we report that the transmembrane chemokine CX3CL1 (fractalkine) is induced in inflamed lymphatic endothelium, both *in vitro* in TNF-α-treated human dermal lymphatic endothelial cells (HDLECs) and *in vivo* in a mouse model of skin hypersensitivity. However, unlike blood endothelial cells, which express predominantly transmembrane CX3CL1 as a leukocyte adhesion molecule, HDLECs shed virtually all CX3CL1 at their basolateral surface through matrix metalloproteinases. We show for the first time that both recombinant soluble CX3CL1 and endogenous secreted CX3CL1 promote basolateral-to-luminal migration of DCs across HDLEC monolayers *in vitro*. Furthermore, we show *in vivo* that neutralising antibodies against CX3CL1 dramatically reduce allergen-induced trafficking of cutaneous DCs to draining lymph nodes as assessed by FITC skin painting in mice. Finally, we show that deletion of the CX3CL1 receptor in *Cx3cr1^−/−^* DCs results in markedly delayed lymphatic trafficking *in vivo* and impaired translymphatic migration *in vitro*, thus establishing a previously unrecognised role for this atypical chemokine in regulating DC trafficking through the lymphatics.

## Introduction

Trafficking of dendritic cells (DCs) from tissues to draining lymph nodes is required to maintain normal immune surveillance and peripheral tolerance (Scheinecker et al., 2002; Steinman et al., 2003). Following infection or the induction of inflammation, bacterial endotoxins and pro-inflammatory cytokines induce maturation of DCs from phagocytes to professional antigen-presenting cells and migration from the tissue into afferent lymphatic capillaries (Steinman et al., 2003). This transmigration across the lymphatic endothelium is one of the most crucial steps in initiating an immune response. It is well documented that the lymphatic endothelial-expressed chemokine CCL21 is required for chemotaxis of DCs to lymphatic vessels during both resting conditions and inflammation, through binding its cognate receptor CCR7 (Förster et al., 1999; Gunn et al., 1999; Ohl et al., 2004; Saeki et al., 1999). Indeed, chemokine-directed, integrin-independent migration, a process termed amoeboid movement, has been proposed as the main mechanism controlling lymphatic entry of DCs, at least in uninflamed tissue (Lämmermann et al., 2008). However, upon the onset of inflammation, when increased production of CCL21 in activated lymphatic endothelium (Johnson and Jackson, 2010; Martín-Fontecha et al., 2003) is accompanied by induction of leukocyte adhesion molecules, including ICAM-1 and VCAM-1 (Johnson et al., 2006), DCs can transmigrate through conventional integrin-dependent mechanisms.

It is becoming increasingly clear that CCL21 is not the only chemokine that controls leukocyte trafficking in the lymphatics. Monocyte-derived DCs have been shown to use CCR8 in addition to CCR7, during migration from the tissues to lymph nodes (Qu et al., 2004). In addition, a role for CXCR4 and its ligand CXCL12 (SDF-1) has been reported for lymphatic migration of both dermal DCs and Langerhans cells (LCs) in skin hypersensitivity responses (Kabashima et al., 2007). Interestingly, the chemotactic effects of CXCL12 and CCL21 were reported to be non-additive, indicating either a sequential mode of action or multiple chemokine redundancy. In this present study, we sought to identify further chemokines that might contribute to lymphatic entry, focusing primarily upon CX3CL1 (fractalkine).

CX3CL1 is expressed in a number of cell types including neurons, intestinal epithelium and activated vascular endothelium (Bazan et al., 1997; Pan et al., 1997) and is structurally distinct from other chemokines in several respects. Unlike most chemokines, CX3CL1 is synthesised as a large, type I integral membrane protein of 373 amino acids, comprising an extracellular domain containing a novel CxxxC chemokine motif and an extended mucin-like stalk (Bazan et al., 1997). This membrane-anchored form of CX3CL1 was originally shown to induce tight, shear-resistant endothelial adhesion of leukocytes in a manner that was apparently independent of integrin and Gi protein activation (Bazan et al., 1997; Fong et al., 1998; Haskell et al., 1999; Imai et al., 1997; Viemann et al., 2004). Subsequently however, soluble forms of CX3CL1 generated by proteolytic cleavage with the disintegrin and metalloproteinases ADAM10 and ADAM17 were shown to promote conventional chemotaxis (Garton et al., 2001; Hundhausen et al., 2003). The functional differences between membrane-bound and soluble CX3CL1, and their respective roles in inflammation remain obscure (Garton et al., 2006; Goda et al., 2000; Umehara et al., 2001).

The sole receptor for CX3CL1 is CX3CR1, which is widely expressed by leukocytes, including CD14^+^ cells of the monocyte-macrophage DC lineage and subsets of tissue-resident DCs and epidermal Langerhans cells (Geissmann et al. 2010; Imai et al., 1997; Jung et al., 2000). In addition to supporting leukocyte extravasation from the blood (Rao et al., 2007), the CX3CL1–CX3CR1 axis has been implicated in pathogenesis of inflammatory diseases such as atherosclerosis, rheumatoid arthritis and renal fibrosis – seemingly by supporting monocyte recruitment and exacerbating tissue damage (Moatti et al., 2001; Tacke et al., 2007; White and Greaves, 2012). Curiously, CX3CL1 was also shown to promote extension of transepithelial dendrites by monocytes and/or DCs within intestinal lamina propria, for the purpose of antigen sensing in the intestinal lumen (Muehlhoefer et al., 2000). However, the significance of the CX3CL1–CX3CR1 axis for control of DC migration in the lymph has yet to be established. Although the original characterisation of constitutive CX3CR1-knockout mice concluded no apparent defects in immune function, the specific role of CX3CL1 and its receptor in regulating lymphatic trafficking was not explored in detail (Jung et al., 2000). Here, we show that CX3CL1 is actively secreted by lymphatic endothelial cells in response to inflammation and that soluble rather than membrane-anchored chemokine promotes DC migration towards lymphatic vessels and lymphatic endothelial transmigration, both *in vitro* and *in vivo*.

## Results

### CX3CL1 is dramatically induced in HDLECs after stimulation with pro-inflammatory cytokines

**Fig. 1.**
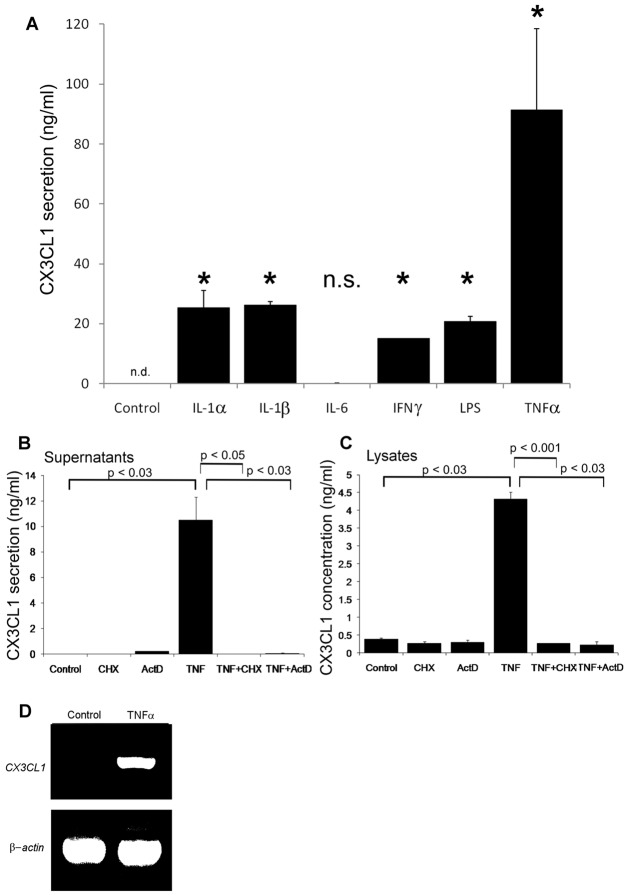
**Induction of CX3CL1 expression in activated lymphatic endothelial cells.** Levels of CX3CL1 secreted from HDLECs stimulated for 24 hours with individual pro-inflammatory cytokines (A) and in the supernatant (B) and detergent lysates (C) of HDLECs stimulated for 24 hours with TNF-α in the presence of actinomycin D (ActD) or cycloheximide (CHX), assessed by ELISA (mean ± s.e., *n* = 3). (D) Comparison of *CX3CL1* mRNA levels in resting and TNF-α-treated (24 hours) HDLECs by RT-PCR with β-actin as a control. Representative data from one experiment of three are shown in each case, *P<0.03 in comparison with CX3CL1 from unstimulated cells.

To investigate expression of CX3CL1 in lymphatic endothelium, early passage human dermal lymphatic endothelial cells (HDLECs) (supplementary material Fig. S1), typically three passages from resection, were cultured in the presence or absence of a panel of pro-inflammatory cytokines and assayed by ELISA for soluble and cell-associated chemokine, in tissue culture supernatant and detergent lysates, respectively. Resting HDLECs were found to secrete little if any CX3CL1 (Fig. 1A–C), whereas stimulation with TNF-α induced a dramatic increase (typically 100 ng/ml). By comparison, IL-1α, IL-1β, IFN-γ and lipopolysaccharide (LPS) induced more modest upregulation (15–30 ng/ml) and IL-6 had no effect. Similar degrees of induction of cell-associated CX3CL1 were observed when assessed in detergent-lysed HDLECs (Fig. 1C and data not shown). To characterise the mode of regulation, we compared CX3CL1 levels in HDLECs stimulated with TNF-α, either alone or in the presence of the RNA polymerase inhibitor actinomycin D or the protein synthesis inhibitor cycloheximide. As shown in Fig. 1B and C, both agents completely suppressed CX3CL1 production, consistent with transcriptional regulation as opposed to mobilisation of pre-packaged chemokine. This was supported by reverse transcriptase PCR analyses, which detected abundant *CX3CL1* mRNA from TNF-α-treated but not control HDLECs (Fig. 1D).

### Membrane-anchored CX3CL1 is immediately shed from the surface of activated HDLECs

**Fig. 2.**
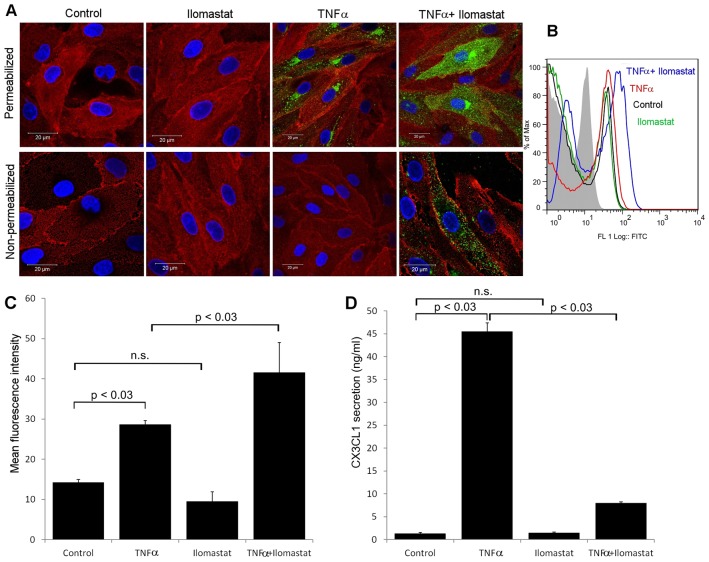
**Rapid shedding of CX3CL1 from the surface of activated HDLECs.** (A) Dual-immunofluorescence staining of control and TNF-α-stimulated (24 hours) HDLECs for CX3CL1 (green) and podoplanin (red) to determine the effect of Ilomastat on intracellular and cell surface CX3CL1 in saponin-permeabilised and non-permeabilised HDLEC monolayers, respectively. Nuclei were counterstained with DAPI; magnification: 630×. FACS histograms (B) and derived mean fluorescence values (C) for cell surface CX3CL1 levels in control HDLECs and HDLECs stimulated with TNF-α in the presence or absence of Ilomastat. Levels of CX3CL1 in supernatants from cells in B and C were also assessed by ELISA (D). Representative data are the means ± s.e. (*n* = 3) from one experiment of three.

To further confirm induction of CX3CL1 in activated HDLECs, intact control and TNF-α-stimulated cells were imaged after staining with fluorescently labelled antibodies. Curiously, no CX3CL1 was detected on the cell surface, regardless of whether HDLECs were stained as adherent monolayers or detached, as single-cell suspensions (Fig. 2A,B). Significantly, however, permeabilisation with saponin revealed diffuse intracellular CX3CL1 staining in a proportion of TNF-α-stimulated cells (Fig. 2A), indicating either that the protein is not targeted to the plasma membrane or that it traffics to the plasma membrane and is then shed rapidly. To distinguish between these possibilities, stimulation with TNF-α was repeated in the presence or absence of the broad-range matrix metalloproteinase inhibitor Ilomastat (GM6001) to block sheddase activity. As shown in Fig. 2A–C and supplementary material Fig. S2, the inclusion of inhibitor led to significant accumulation of CX3CL1 at the cell surface of individual HDLECs and also reduced the amount secreted into the culture medium (Fig. 2D). These results indicate that the soluble form of CX3CL1 is shed from the HDLEC surface almost immediately after synthesis.

### CX3CL1 shedding is mediated by ADAM10 and ADAM17

**Fig. 3.**
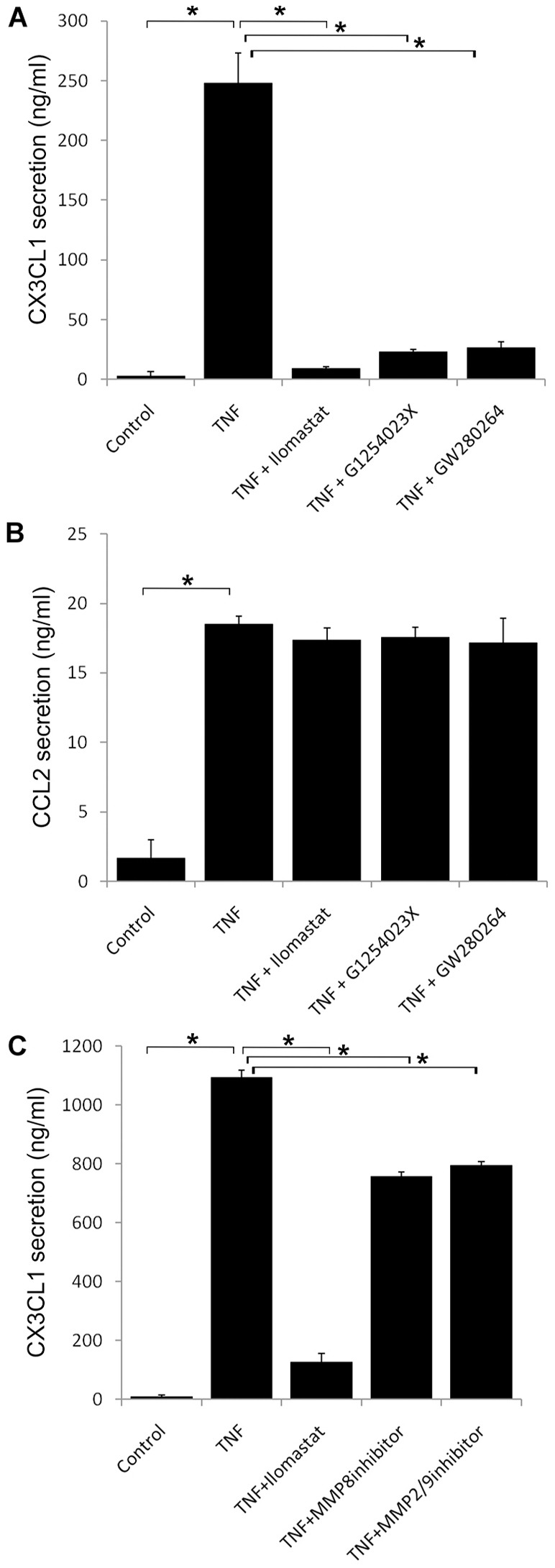
**Shedding of CX3CL1 involves cleavage by ADAM10 and ADAM17.** The amount of CX3CL1 (A) and CCL2 (B) shed from HDLECs cultured in medium alone (control) or in the presence of TNF-α for 24 hours, with either Ilomastat or the ADAM10/17 inhibitors GI254023X and GW280264X, assessed by ELISA. (C) The amount of CX3CL1 shed from HDLECs under similar conditions to A and B but with inhibitors to MMP8 and MMP2/9. Data are the means ± s.e. (*n* = 3) from one representative experiment of three; *P<0.03.

Next, we sought to identify the metalloproteinases that mediate CX3CL1 shedding. Previously, ADAM10, ADAM17 and MMP2 have been shown to mediate both constitutive and activation-induced shedding of CX3CL1 from vascular endothelial cells (HUVECs), transfected fibroblasts and hepatic stellate cells (Bourd-Boittin et al., 2009; Garton et al., 2001; Goda et al., 2000; Hundhausen et al., 2003). Incubation of TNF-α-stimulated HDLEC monolayers with the ADAM10 and ADAM17 inhibitors GI254023X and GW280264X revealed a concentration-dependent inhibition of CX3CL1 secretion (Fig. 3A and supplementary material Fig. S3). Importantly, neither inhibitor significantly affected HDLEC viability or secretion of the cleavage-independent chemokine CCL2 (MCP-1), which served as a control (Fig. 3B). In addition, the MMP2 and MMP9 inhibitor II and MMP8 inhibitor I also reduced CX3CL1 shedding although to a lesser extent (∼25%) than GI254023X and GW280264X (Fig. 3C), suggesting that ADAM10 and ADAM17 are the prime sheddases involved in endogenous secretion of CX3CL1 from HDLEC.

### CX3CL1 is shed predominantly from the basolateral surface of HDLEC monolayers

**Fig. 4.**
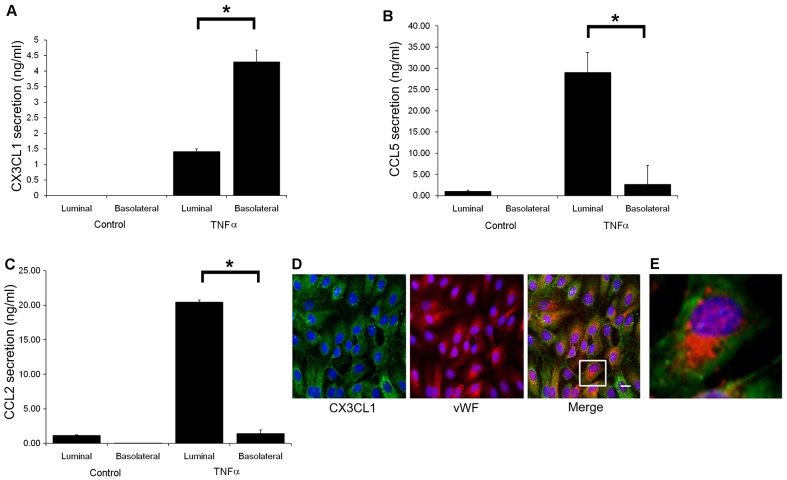
**CX3CL1 is selectively secreted from the basolateral surface of activated HDLECs.** Amounts of CX3CL1 (A), CCL5 (B) and CCL2 (C) secreted from the basolateral and luminal surfaces of control and TNF-α-stimulated HDLEC monolayers cultured for 24 hours on Fluoroblok Transwell inserts, as assessed by ELISA. Data are the mean ± s.e. (*n* = 3) from one representative experiment of three; *P<0.03. (D) Dual immunofluorescence staining for CX3CL1 (green) and vWF (red) in saponin-permeabilised TNF-α-treated HDLECs indicates lack of colocalisation with Weibel–Palade bodies. Nuclei were counterstained with TOPRO-3. Scale bar: 10 µm. (E) Digital zoom of indicated cell in D.

To determine whether secretion of CX3CL1 was polarised, HDLEC monolayers cultured on Transwell inserts were stimulated with TNF-α and chemokine secretion measured from both luminal and basolateral surfaces (i.e. top and bottom chambers) by ELISA. Strikingly, CX3CL1 was secreted predominantly (>70%) from the basolateral surface (Fig. 4A). By contrast, secretion of the chemokines CCL5 (RANTES) and CCL2, both of which are induced in activated HDLECs (Johnson et al., 2006), was almost exclusively luminal (>90%) (Fig. 4B,C). Furthermore, dual-fluorescence staining of permeabilised HDLECs with the marker von Willebrand Factor (vWF) showed essentially no CX3CL1 within Weibel–Palade bodies (Fig. 4D,E) – the organelles associated with luminal secretion of chemokines such as CXCL8 (IL-8) in blood endothelial cells (Wolff et al., 1998). These findings suggest that *in vivo*, lymphatic endothelial cell (LEC)-derived CX3CL1 functions perivascularly, influencing events directly adjacent to, rather than on, the inner surface of lymphatic vessels. This would distinguish CX3CL1 from chemokines such as CCL2 that are secreted in lymphatic vessels for remote functions in downstream lymphoid tissue (Palframan et al., 2001).

### CX3CL1 is expressed in inflamed lymphatic vessels *in vivo*

**Fig. 5.**
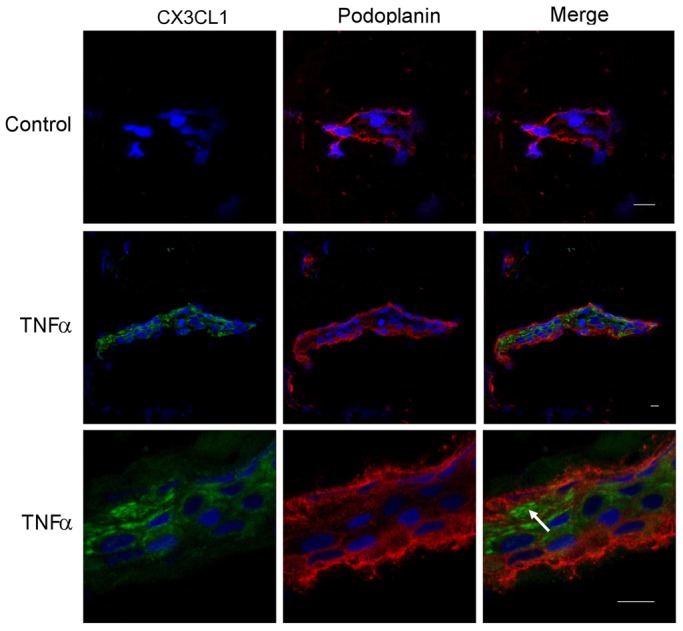
**Visualisation of CX3CL1 in lymphatic vessels of TNF-α-activated human dermis.** Dual immunofluorescence staining for CX3CL1 (green) and podoplanin (red) in permeabilised frozen sections (<4 µm) of human dermis, either freshly resected (control) or cultured for 24 hours in the presence of TNF-α. Nuclei were counterstained with TOPRO-3. Arrow indicates typical intracellular location of CX3CL1. Scale bars: 10 µm.

**Fig. 6.**
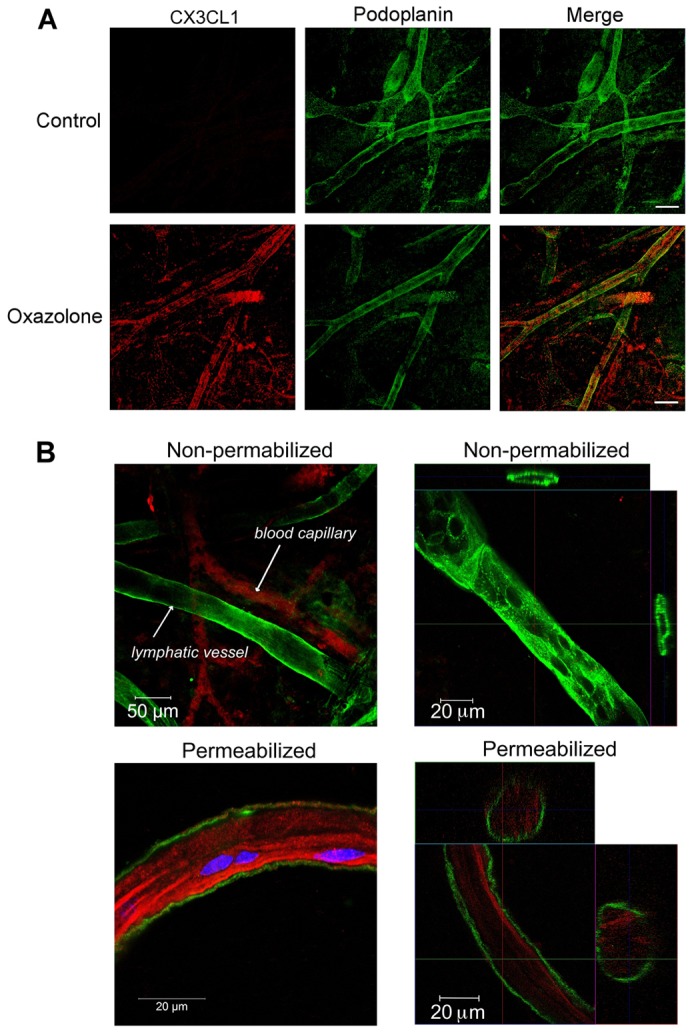
**Visualisation of CX3CL1 in dermal lymphatics of contact-hypersensitised mouse skin.** (A) Whole-mount sections of control contralateral uninflamed ear skin and inflamed ear skin from mice subjected to topical oxazolone-stimulated skin hypersensitivity, permeabilised and stained for CX3CL1 (red) and podoplanin (green). Scale bars: 100 µm. Representative images from four mice are shown. (B) Whole-mount sections of inflamed ear skin were prepared in either the absence or presence of Triton X-100 (non-permeabilised and permeabilised, respectively), immunostained as above and imaged by confocal microscopy as a *Z*-series. Single planes of view are shown in left-hand panels; orthogonal views of different vessels in right-hand panels.

To assess CX3CL1 expression in intact lymphatic endothelium, we performed immunostaining of frozen sections from both freshly resected and TNF-α-treated human dermis after permeabilisation (Fig. 5). No CX3CL1 was detected within resting podoplanin^+^ lymphatics in normal skin. By contrast, high levels were detected in lymphatic vessel endothelium of TNF-α-treated skin, in intracellular patches that were visible only after permeabilisation of the sections. Significantly, no CX3CL1 appeared to colocalise with podoplanin at the surface of lymphatic endothelium. Next, we investigated CX3CL1 induction in lymphatic endothelium within oxazolone contact-hypersensitised skin from both BALB/c and C57Bl/6 mice, and in explanted TNF-α-treated mouse dermis. As shown in Fig. 6A and supplementary material Fig. S4, CX3CL1 staining was detected in podoplanin^+^ lymphatic vessels in both cases, but only when tissue had been permeabilised with Triton X-100 before staining. By contrast, CX3CL1 could be readily detected on podoplanin^−^ blood capillaries in non-permeabilised tissue (Fig. 6B). Furthermore, CX3CL1 in inflamed lymphatic endothelium could be seen in a perinuclear pattern, although in some sections, residual amounts also appeared to be present in the vessel lumen (Fig. 6B, bottom left panel). Little could be detected near the subendothelial surfaces of lymphatic vessels, in contrast to reports of this location for CCL21 (Tal et al., 2011; Weber et al., 2013) and consistent with the fact that CX3CL1 does not bind heparin and acts as a fluid phase rather than an immobilised chemokine (Patel et al., 2001). In addition to CX3CL1, oxazolone sensitisation also led to upregulation of CCL5 and CCL20, which were concentrated around podoplanin^+^ lymphatic vessels, and CCL2, which had a more diffuse pattern within the tissue (supplementary material Fig. S5). These results show for the first time that CX3CL1 is induced as part of a pleiotropic response by lymphatic endothelium to inflammation in both mice and humans, and confirm that the molecule is stored intracellularly in preparation for secretion, rather than retained at the cell surface, both *in vitro* and *in vivo*.

### CX3CL1 promotes polarised transmigration of DCs across lymphatic endothelium

**Fig. 7.**
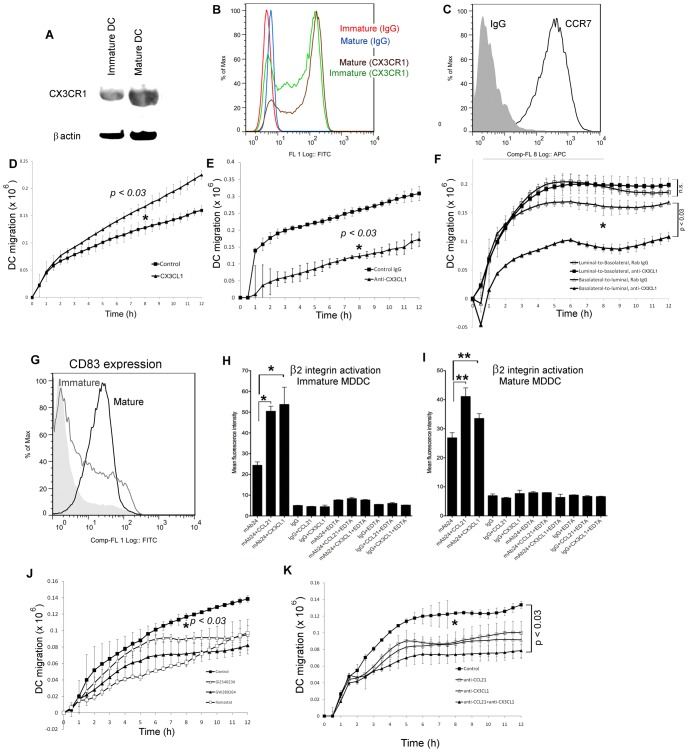
**CX3CL1 promotes DC transmigration across resting and activated HDLECs *in vitro*.** Levels of CX3CR1 expression in immature and LPS-matured MDDCs as assessed by western blotting (with inclusion of β-actin as loading control) (A) and flow cytometry (B). Expression of CCR7 was also detected (C) and maturation was assessed by expression of CD83 (G). Number of fluorescently labelled MDDCs transmigrating resting HDLECs, incubated either alone or with recombinant human CX3CL1 (D) and TNF-α-stimulated HDLECs incubated either with chemokine-neutralising antibodies or irrelevant IgG controls (E,F,K) or ADAM and metalloproteinase inhibitors (J). Activation of β2 integrin was assessed through binding of mAb24 to immature (H) and mature (I) MDDCs; *P<0.03, **P<0.015. Mouse IgG1 was included as an isotype-matched control. EDTA chelates divalent cations and inhibits integrin activation, thus indicating specificity of mAb24 binding. All data represent the mean ± s.e. (*n* = 4) from one representative experiment that was repeated two to five times.

The observed induction and polarised secretion of CX3CL1 is clearly suggestive of a role in directing leukocyte chemotaxis and/or entry to lymphatic vessels. To explore this possibility, we focused on trafficking of DCs, the major migratory leukocyte population in afferent lymph, using human blood monocyte-derived DCs (MDDCs) induced to mature by treatment with LPS as a model. Both immature and mature MDDCs express significant levels of CX3CL1 receptor, CX3CR1, assessed by quantitative western analysis and flow cytometry (Fig. 7A,B), in addition to the essential lymphoid chemokine receptor CCR7 (Fig. 7C). We first measured the capacity of exogenously added CX3CL1 to stimulate basolateral-to-luminal migration of fluorescently labelled MDDCs across monolayers of HDLECs plated on the under-surface of Transwell FluoroBlok membranes (Johnson et al., 2006). As shown in Fig. 7D, addition of CX3CL1 to the upper chamber significantly increased both the number of transmigrating MDDC and the rate of transmigration compared with unsupplemented medium. Next, to assess the contribution of endogenous, activation-induced CX3CL1 to DC transmigration, we carried out assays using TNF-α-stimulated HDLECs, in the presence of either a CX3CL1 neutralising antibody or irrelevant rabbit IgG added to the upper chamber. As shown in Fig. 7E, neutralising antibody reduced DC transmigration by ∼50%, confirming the proposed role for HDLEC-expressed CX3CL1 in mediating inflammatory MDDC transit *in vitro*. Notably, addition of CX3CL1 neutralising antibodies to the upper chamber did not inhibit migration of MDDCs in the reverse luminal-to-basolateral direction (Fig. 7F), consistent with the observed polarity of CX3CL1 secretion from the basolateral face of activated HDLECs (Fig. 4A). Importantly, blockade of CX3CL1 shedding by treatment of HDLECs with ADAM10 and ADAM17 inhibitors, or Ilomastat, led to the same reduction in MDDC transmigration as the CX3CL1 neutralising antibody (Fig. 6J). Hence, we conclude it is the secreted rather than the membrane-bound form of CX3CL1 that is active in mediating DC transmigration.

Given that activated HDLECs also secrete CCL21 and that neutralising antibodies to CCL21 partially block DC transmigration across TNFα-treated HDLEC *in vitro* (Johnson and Jackson, 2010), we investigated the consequences of imposing a dual block with CCL21 and CX3CL1 neutralising antibodies in the upper chamber of the Transwell. The results (Fig. 7K) show that the effect of the antibodies was not additive, yielding less than complete inhibition of DC transmigration. Hence, it is possible that CX3CL1 and CCL21 act sequentially in activated lymphatic endothelium and that additional as yet unidentified chemokines contribute to transmigration. Furthermore, our previous studies have shown that when G-protein-coupled chemokine receptors are inhibited by pertussis toxin, 15–20% of input DCs can still transmigrate across lymphatic endothelium, i.e. in a chemokine-independent manner (Johnson and Jackson, 2010).

Finally, we evaluated whether the mode of action of CX3CL1 in DC transmigration involves activation of β2 integrin, similar to that documented for CCL21 (Johnson and Jackson, 2010). Accordingly, we used mAb24, an antibody that selectively binds an active conformation of the β2 integrin subunit (Dransfield et al., 1992) and compared reactivity with MDDCs exposed to either CX3CL1 or CCL21. CX3CL1 exposure increased mAb24 reactivity of both immature and mature MDDCs, to levels that were comparable to those of CCL21 (Fig. 7G–I). However, CX3CL1 had no significant effect on ICAM-1 or VCAM-1 levels in HDLECs within the 4 hour time-frame of the *in vitro* transmigration assays (supplementary material Fig. S6). These results demonstrate for the first time that soluble CX3CL1 secreted from TNFα-stimulated lymphatic endothelium can activate β2 integrin adhesion in DCs and direct their basolateral-to-luminal transmigration.

### CX3CL1 promotes lymphatic trafficking of DCs in a mouse model of skin inflammation *in vivo*

**Fig. 8.**
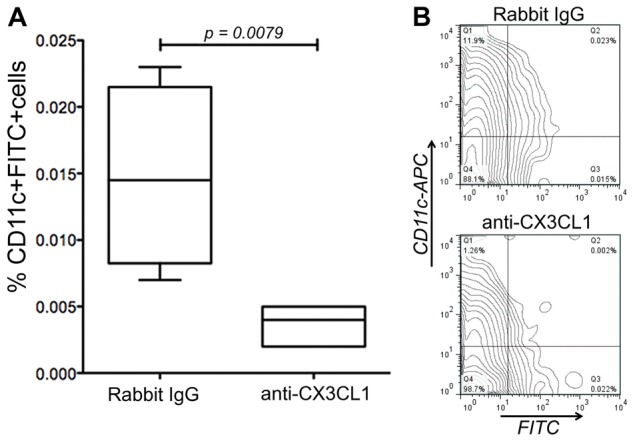
***In vivo* trafficking of cutaneous DCs through afferent lymphatics is dependent upon CX3CL1.** Recovery of FITC^+^ CD11c^+^ skin DCs in the draining lymph nodes, 24 hours after FITC skin painting of oxazolone-sensitised mice that received prior injection of neutralising antibodies against CX3CL1 or control rabbit IgG. Data represent the mean recoveries ± s.e. (*n* = 5) from one experiment of two (A), with representative flow cytometry contour plots (B).

Having established inflammation-induced expression of CX3CL1 in primary LEC and a role in transmigration of DCs *in vitro*, we next assessed the role of CX3CL1 in lymphatic trafficking of endogenous DCs *in vivo*, using the well-characterised oxazolone-induced skin-hypersensitivity model mentioned above (Fig. 6). Oxazolone-treated mice were injected with neutralising antibodies against CX3CL1, and migration of cutaneous DCs following FITC skin painting was measured by flow cytometry of disrupted skin-draining lymph nodes. The results showed that after 24 hours, block of CX3CL1 suppressed lymph node trafficking of FITC^+^, CD11c^+^ DCs by >70% when compared with rabbit IgG controls (Fig. 8A,B). As expected, skin-derived DCs recovered from draining lymph nodes of all mice were found to express both CCR7 and CX3CR1 (data not shown).

### Genetic deletion of CX3CR1 impairs lymphatic migration of DCs *in vivo*

**Fig. 9.**
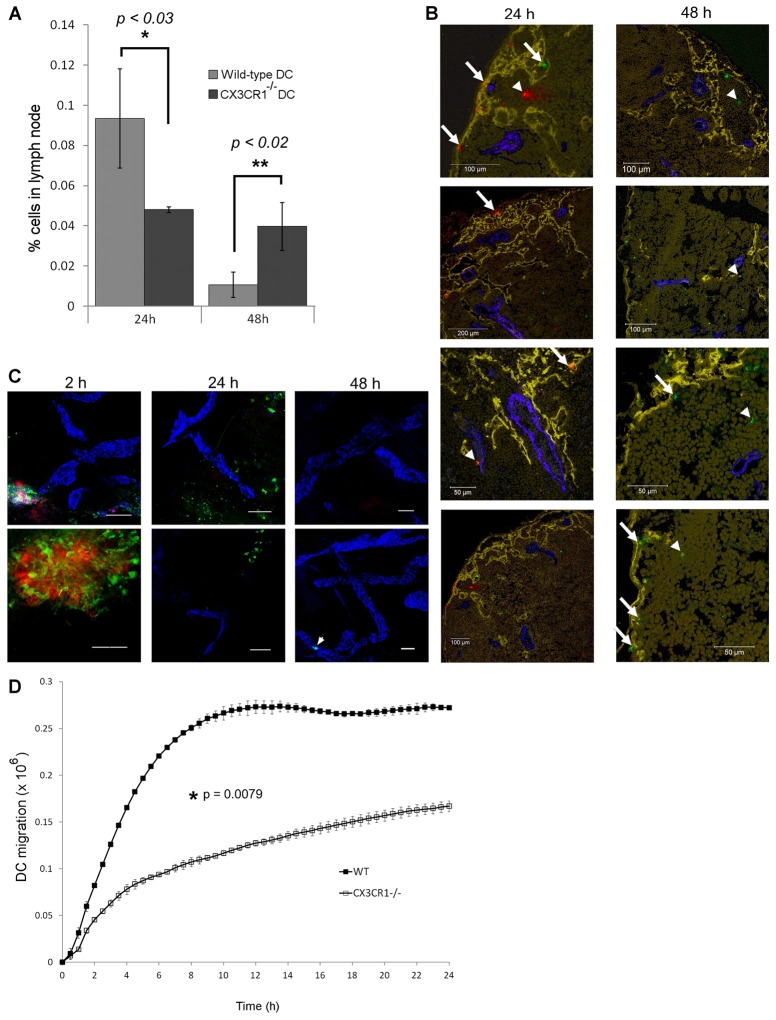
**CX3CR1 is required for efficient DC trafficking through inflamed skin lymphatics.** (A) Number of wild-type (Q-tracker525 labelled) and *Cx3cr1^−/−^* (Q-tracker655 labelled) BMDCs arriving in draining lymph nodes 24 or 48 hours after co-injection of a 50∶50 mixture into the skin of topical oxazolone-sensitised C57Bl/6 mice. Data represent the means ± s.e. (*n* = 3) from one representative experiment of three; *P<0.001, **P<0.0079. (B) Fluorescent imaging of wild-type (Cell Tracker Red labelled) and *Cx3cr1^−/−^* (Cell Tracker Green labelled) BMDCs in draining lymph nodes at 24 and 48 hours after injection. Frozen sections of lymph nodes were stained with anti-LYVE-1 (yellow) and MECA79 (blue) to label lymphatic vessels or subcapsular sinus and HEVs, respectively. Nuclei were counterstained with TOPRO-3, yellow. Arrows indicate examples of BMDCs in the subcapsular sinus; arrowheads indicate BMDCs in paracortex of lymph nodes. (C) Whole-mount skin sections with LYVE-1^+^ lymphatic vessels (blue) comparing numbers of wild-type (red) and *Cx3cr1^−/−^* (green) adoptively transferred BMDCs remaining at 2, 24 and 48 hours after co-injection. Arrow indicates one of a very few *Cx3cr1^−/−^* BMDCs that remained at 48 hours. Representative images from six mice are shown. Scale bars: 50 µm. (D) Number of fluorescently labelled wild-type and *Cx3cr1^−/−^* DCs transmigrating across TNF-α-stimulated MDLEC monolayers plated on the underside of FluoroBlok inserts. Data represent the means ± s.e. (*n* = 4) from one representative experiment of three.

Cutaneous DCs migrate to lymph nodes almost exclusively via afferent lymphatics, rather than by entering the blood circulation. Hence, CX3CL1 and its receptor must play a role in either the initial entry to or subsequent migration within lymphatics. To address this question, we compared the migration of bone marrow-derived DCs (BMDCs) from wild-type and *Cx3cr1^−/−^* mice, after co-injection into the inflamed skin of topical oxazolone-hypersensitised wild-type mice. To allow discrimination between the cell types, wild-type BMDCs were labelled with Q-tracker655 and *Cx3cr1^−/−^* BMDCs were labelled with Q-tracker525, immediately before adoptive transfer. Importantly, before injection, both cell populations showed similar expression of MHC class II, the DC-selective β2 integrin CD11c, the co-stimulatory molecule CD86 (B7.2) and the lymph migratory dermal DC marker EpCAM (supplementary material Fig. S7). As shown in Fig. 9A, however, the excised cervical lymph nodes revealed marked differences in the migratory properties of wild-type and *Cx3cr1^−/−^* BMDCs. The majority of wild-type BMDCs had migrated within 24 hours, but were largely dispersed by 48 hours, by either exiting in the efferent lymph or undergoing apoptosis *in situ*. By contrast, the *Cx3cr1^−/−^* BMDC population was significantly slower to migrate, with the majority taking 48 hours to reach the same lymph nodes [0.094±0.018% wild-type BMDCs (mean ± s.e.m.) versus 0.048±0.001% *Cx3cr1^−/−^* BMDCs at 24 hours; 0.011±0.003% wild-type BMDCs versus 0.040±0.006% *Cx3cr1^−/−^* BMDCs at 48 hours). Importantly, the total numbers of wild-type and knockout BMDCs recovered from the cervical lymph nodes after 24 hours and 48 hours were almost identical, indicating that CX3CR1-deficient BMDCs experience a delay in migration through afferent lymphatic vessels, rather than undergoing apoptosis. Similar results were also obtained when wild-type and *Cx3cr1^−/−^* BMDCs were injected into separate animals, rather than co-injected (data not shown). These findings indicate for the first time that CX3CL1 functions as an accessory chemokine for DC mobilisation through the lymph *in vivo*.

To further investigate the role of CX3CL1 in DC trafficking, oxazolone-hypersensitised mice were injected intradermally with equal mixtures of wild-type and *Cx3cr1^−/−^* BMDCs labelled with Cell Tracker Red and Cell Tracker Green respectively, to allow fluorescent microscopic imaging of both the draining lymph nodes (Fig. 9B) and the skin injection site (Fig. 9C) at 24 hours and 48 hours after adoptive transfer. This confirmed the delay in trafficking of *Cx3cr1^−/−^* BMDCs in lymph nodes observed by quantitative flow cytometry. Analysis of DCs that had trafficked to lymph nodes revealed no obvious differences between localisation of wild-type and *Cx3cr1^−/−^* populations (Fig. 9B). By contrast, imaging of the skin injection site revealed that *Cx3cr1^−/−^* BMDCs were heavily predominant at 24 hours post injection, visible either as discrete cells or larger clusters (Fig. 9C), despite similar numbers of *Cx3cr1^−/−^* and wild-type BMDCs within the dermis at early time points (2 hours). Significantly, few if any *Cx3cr1^−/−^* BMDCs were seen to accumulate at the basolateral surface of lymphatic vessels in these areas at 24 hours, suggesting that CX3CL1–CX3CR1 influences DC migration towards endothelium rather than endothelial adhesion. By 48 hours, BMDCs were rarely observed within the dermis, the majority of cells having exited the tissue, most likely through the afferent lymphatics.

To verify that the delay in lymphatic trafficking of *Cx3cr1^−/−^* BMDCs *in vivo* reflects an impairment in transendothelial migration, we compared the kinetics of basolateral-to-luminal migration for wild-type and receptor-null DCs *in vitro* using TNF-α-activated mouse dermal lymphatic endothelial cell (MDLEC) monolayers (supplementary material Fig. S8), plated on the underside of Transwell inserts. Over the course of 24 hours, strikingly fewer BMDCs from CX3CR1-deficient mice migrated across the lymphatic endothelium than their wild-type counterparts, and at a consistently slower rate (Fig. 9D). Hence, we conclude that CX3CL1 regulates both migration of DCs towards lymphatic capillaries and also the subsequent transendothelial transit of DCs into the vessels.

## Discussion

Here we present evidence that CX3CL1, the sole member of the atypical transmembrane CX3C chemokine family, is synthesised *de novo* and shed in soluble form by inflamed human and murine LECs. Additionally, we confirm induction of CX3CL1 in intact lymphatic vessels of TNF-α-treated mouse and human dermis, and skin sections from contact hypersensitised mice. Moreover, we demonstrate that secreted CX3CL1 promotes migration of MDDC across activated lymphatic endothelium *in vitro* and that neutralising antibodies against CX3CL1 inhibit lymphatic trafficking of cutaneous DCs to draining lymph nodes *in vivo*. In further corroboration of these findings, we show that targeted disruption of the CX3CL1 axis in BMDCs by deletion of the receptor CX3CR1 delays trafficking from inflamed skin to draining lymph nodes. These results identify a significant new player in the regulation of cell trafficking in the lymphatics during inflammation and attest further to the complexity of what has been considered in the past as merely a passive process.

CX3CL1 is particularly unusual insofar as it can occur both as an integral membrane adhesion protein and a conventional chemokine, generated through controlled cleavage by the disintegrin and metalloprotease enzymes ADAM17 and ADAM10 (Bazan et al., 1997; Garton et al., 2001; Hundhausen et al., 2003; Pan et al., 1997). Although the precise functional differences between the two forms are still obscure, it is clear that interaction of membrane-anchored CX3CL1 with its receptor, CX3CR1, on leukocytes confers shear-resistant binding to blood vascular endothelium in a mechanism that is independent of integrin and Gi protein involvement (Fong et al., 1998; Haskell et al., 1999). By contrast, the secreted form of CX3CL1 has been shown to promote chemotaxis of CX3CR1^+^ monocytes, T cells and NK cells by a conventional mechanism of integrin-mediated adhesion (Goda et al., 2000; Imai et al., 1997; Yoshikawa et al., 2004). Notably, however, it is the membrane-anchored form that predominates in most cells and hence the function of CX3CL1 has been regarded as one of cell adhesion as much as chemotaxis.

An intriguing finding from the studies in this present manuscript was that both cultured LECs and lymphatic endothelium *in vivo* generate almost exclusively the soluble form of CX3CL1, through the action of an ADAM-like sheddase activity, and retain little, if any, of the membrane-anchored form. Previous reports indicate that in other cell types, CX3CL1 can evade proteolytic cleavage by a constitutive clathrin-mediated endocytosis pathway (Huang et al., 2009; Liu et al., 2005). It might be that this pathway operates less efficiently in LECs, or alternatively that they exhibit a greater level of CX3CL1 sheddase activity. Moreover, as the interstitial migration of leukocytes into lymphatic vessels does not have to combat the shear flow that restricts their exit from blood capillaries, there might be a lesser requirement for CX3CL1 as an adhesion receptor, and in contrast to the blood vascular endothelium, the soluble chemokine form of the molecule might be favoured. We noted heterogeneity of CX3CL1 expression within TNFα-stimulated HDLECs, and suspect that this reflects the subtle variability in HDLEC subtype, because these cells are isolated from the skin by immunoselection with LYVE-1 and thus derive from a mixture of both initial and precollector lymphatics. Indeed, we have previously observed similar heterogeneity in expression of VCAM-1 (Johnson et al., 2006).

Another particularly important finding was that CX3CL1 is secreted almost exclusively from the basolateral surface of lymphatic endothelium, where it selectively promotes basolateral-to-luminal transmigration of DCs. This makes CX3CL1 virtually unique within the large repertoire of CC and CXC chemokines synthesised by resting and inflamed LECs, including CCL21, CXCL12 (SDF-1), CCL2 (MCP-1), CCL5 (RANTES), CCL20 (MIP-3α), CXCL2 (GROβ), CXCL5 (ENA78) and CXCL8 (IL-8) that influence trafficking of DCs, monocytes, T cells and neutrophils (Gunn et al., 1999; Johnson et al., 2006; Johnson and Jackson, 2010; Kabashima et al., 2007; Kriehuber et al., 2001; Saeki et al., 1999) (and data not shown), most or all of which are secreted predominantly from the luminal surface. Even CCL21, which is well-documented as being essential for lymphatic entry, is biased mainly (∼70%) towards luminal secretion (Kriehuber et al., 2001). The fate of luminally secreted CCL21 is at present unclear, although it could be conveyed away from the initial lymphatics by afferent lymph flow and hence play a role downstream in the lymph node, as has been shown for CCL2 (Palframan et al., 2001). By contrast, basolaterally secreted CCL21 is sequestered on the basement membrane of lymphatic vessels, where it is complexed with collagen IV at ‘entry portals’, facilitating the docking and transmigration of DCs (Tal et al., 2011). On the basis of its highly polarised secretion, we propose that CX3CL1 preferentially influences events in the subendothelial zone. For example, during inflammation, when lymph flow is retarded, the accumulation of CX3CL1 might establish local chemokine gradients that attract DCs towards the basolateral surface of the lymphatic vessel, before CCL21 guidance.

A role for CX3CL1 in lymphatic trafficking of endogenous DCs was demonstrated by measuring FITC^+^/CD11c^+^ cells in draining lymph nodes 24 hours after FITC skin painting. CX3CL1 blockade by neutralising antibodies suppressed lymph node trafficking of FITC^+^/CD11c^+^ cells by >70% when compared with IgG controls. In addition to impairing DC entry to lymphatics, it is also possible that CX3CL1 neutralising antibodies might have blocked recruitment of monocytes from the blood in these experiments, thus reducing the number of monocyte-derived DCs entering the dermis. However, 24 hours after the onset of inflammation, the major population trafficking to the lymph nodes is more likely to be dermal DCs than newly recruited and differentiated monocyte-derived DCs.

Most significantly, we observed in our adoptive-transfer experiments that genetic deletion of *Cx3cl1* in DCs impeded trafficking from the inflamed dermis of oxazolone hypersensitised mice, delaying their transit to skin-draining cervical lymph nodes by ∼24 hours compared with wild-type cells. Indeed, the transient nature of this inhibition might also explain how the influence of CX3CL1 on DC trafficking was overlooked during the original characterisation of global *Cx3cr1^−/−^* knockout mice (Jung et al., 2000). Also, the authors of the previous study made indirect measurements of DC migration rather than quantifying the kinetics of trafficking using fluorescently labelled DCs, as we have done here. Importantly, in our experiments, total numbers of wild-type and *Cx3cr1^−/−^* BMDCs recovered from the cervical lymph nodes after 24 hours and 48 hours were almost identical. Hence, the effects of disrupting CX3CR1–CX3CL1 cannot be explained by an increase in DC apoptosis.

The delayed trafficking of CX3CR1-deficient DCs also contrasts with the sustained reduction in lymphatic entry and lymph node migration of DCs that has been reported after disruption of the key CCL21–CCR7 axis in CCR7^−/−^ mice (Ohl et al., 2004) or following inhibition of the CXCL12–CXCR4 axis (Kabashima et al., 2007). This probably reflects differences in the sites of action of each chemokine, as well as their relative positions in the functional hierarchy. CCL21 has several actions, promoting not only interstitial chemotaxis of DC by amoeboid movement but most notably transendothelial migration by β2 integrin activation and intraluminal crawling under both resting and inflammatory conditions (Johnson and Jackson, 2010; Tal et al., 2011). Such control over the rate-limiting step in DC transmigration therefore places the CCL21–CCR7 axis in a dominant position in the hierarchy of ‘lymphatic’ chemokines and might explain why CX3CL1–CX3CR1 is insufficient to maintain DC trafficking in the absence of CCL21–CCR7. We propose that CX3CL1 and CCL21 act at sequential stages during the migratory cascade, consistent with our finding that both CX3CL1 and CCL21 neutralising antibodies inhibit DC transmigration when added singly, but fail to yield additive effects on blockade when combined together. CX3CL1 does not contain a basic GAG-binding tract as is found in other matrix-sequestered chemokines such as CCL21 and CCL5 and consequently is not sequestered in tissue extracellular matrix (Patel et al., 2001). Thus, CX3CL1 could be expected to play a role in fluid-phase chemotaxis, rather than haptotaxis, and provide initial guidance to DCs towards the lymphatic capillaries, before the stage at which immobilised CCL21 aids in docking and transmigration (Tal et al., 2011). Moreover, because CX3CL1 is induced strictly in response to inflammation, we conclude that the CX3CL1–CX3CR1 axis, unlike CCL21–CCR7, guides DC trafficking under inflammatory conditions only.

Overall, our findings demonstrate induction of CX3CL1 secretion in inflamed lymphatic endothelium and a novel role for this atypical chemokine in regulating trafficking of DCs from inflamed skin through afferent lymphatic vessels. It is now clear that this complex process is far from passive, and CX3CL1 is induced as part of a wider pleiotropic response by the lymphatic endothelium to inflammation.

## Materials and Methods

### Human and animal studies

All studies using human tissue were approved by the Oxford Regional Ethics Committee. All animal studies were performed with appropriate UK Home Office licences according to established institutional guidelines.

### Cytokines, chemokines and growth factors

Recombinant human and mouse proteins were from R&D Systems and used at the following concentrations: IL-1α and IL-1β, 1 ng/ml; IL-6, 20 ng/ml; IFN-γ, 100 ng/ml; human TNFα, 2 ng/ml; mouse TNFα, 100 ng/ml; human IL-4, 10 ng/ml; human GM-CSF, 50 ng/ml; mouse IL-4, 20 ng/ml; mouse GM-CSF, 20 ng/ml; and human CX3CL1 (chemokine domain), 25 µg/ml. LPS (1 µg/ml) from *Salmonella Abortus* was from Sigma-Aldrich.

### Antibodies

Rabbit anti-CX3CL1 and goat anti-CCL21 neutralising antibodies were from AMS Biotechnology and R&D Systems, respectively. Other antibodies used were mouse anti-human CX3CL1 (R&D Systems), rat anti-CX3CR1 (AMS Biotechnology), rabbit anti-human podoplanin (Fitzgerald Industries International), rabbit anti-mouse podoplanin, clone 8.1.1 (Developmental Studies Hybridoma Bank, University of Iowa, Iowa City, IA), mouse anti-human ICAM-1 (Chemicon), mouse anti-human VCAM-1 (BD Pharmingen), mouse anti-human E-selectin (Developmental Studies Hybridoma Bank), sheep anti-human von Willebrand factor (vWf), (Serotec) and rat anti-mouse EpCAM (eBioscience). Goat anti-mouse CCL2, goat anti-mouse CCL5 and goat anti-mouse CCL20 were from R&D Systems. Directly conjugated anti-mouse CD86-PE, CD11c-APC and CCR7-PE were from BD Pharmingen. All anti-LYVE-1 antibodies were generated previously (Banerji et al., 1999; Johnson et al., 2006; Prevo et al., 2001). Isotype-matched antibodies were from Sigma-Aldrich or R&D Systems.

### Cells

Primary HDLECs and MDLECs were prepared from freshly resected skin samples by immunoselection with LYVE-1 mAb and MACS beads (Miltenyi Biotec), and cultured as previously described (Johnson et al., 2006). MDDCs were obtained from healthy donors and generated as previously described (Johnson et al., 2006). MDDCs were fluorescently labelled using Cell Tracker Green (Invitrogen), following the manufacturer's protocol. BMDCs were extracted from tibia and fibula bone marrows of euthanised C57Bl/6 and *Cx3cr1^−/−^* mice, passed through a 70 µm cell strainer (BD Biosciences) and cultured for 7 days in RPMI with 10% FCS and supplemented with GM-CSF and IL-4. Non-adherent cells were selected for MHC class II expression by MACS beads (Miltenyi Biotec) and then either labelled with Q-tracker655 or Q-tracker525 (Molecular Probes, Invitrogen), or with Cell Tracker Green or Cell Tracker Red (Invitrogen), following the manufacturer's protocol.

### Inhibitors

Ilomastat (GM6001) was purchased from Calbiochem and used at 2.5 µM. ADAM10 and ADAM17 inhibitors GW280264X and GI254023X were a kind gift from Dr Andreas Ludwig, Aachen University, Germany). MMP2 and MMP9 inhibitor II and MMP8 inhibitor I were purchased from Calbiochem and used at 1 µM. Actinomycin D and cycloheximide were purchased from Sigma-Aldrich and used at 10 µg/ml and 20 µg/ml, respectively.

### Immunofluorescence antibody staining of cells and tissues

Cultured HDLECs and MDLECs were fixed in paraformaldehyde (1% w/v in PBS, pH 7.4; PFA-PBS); incubated with the appropriate primary antibodies in PBS with 10% (v/v) FCS and 1% (w/v) BSA, with or without prior cell permeabilisation in 0.2% saponin for 45 minutes at room temperature. Alexa Fluor 488, Alexa Fluor 568, Alexa Fluor 647 conjugates and either TOPRO-3 or DAPI nuclei stain (Molecular Probes, Invitrogen) were used for secondary detection before fixing in PFA-PBS, mounting in Vectashield-DAPI (Vector Laboratories) and viewing under Bio-Rad Radiance 2000 or Zeiss LSM780 confocal microscopes, using either a Plan-Apochromat 10× /0.3 DIC M27 (total magnification: 100×) or Plan-Apochromat 63× /1.4 oil (total magnification: 630×, resolution: 0.24 µm). Whole-mount tissue staining was carried out as described previously (Johnson et al., 2006), either permeabilising tissue with 0.1% (v/v) Triton X-100 in PBS or washing in PBS alone, to observe non-permeabilised tissue. Frozen sections of lymph node were prepared by cryostat, fixed for 5 minutes in ice-cold acetone then incubated with rat antibodies against mouse LYVE-1 [clone C1/8 (Johnson et al., 2007)] directly conjugated with Alexa Fluor 647 and biotinylated MECA79 in PBS with 10% (v/v) FCS and 1% (w/v) BSA. Streptavidin conjugated to Pacific Blue (Molecular Probes, Invitrogen) was used for secondary detection and nuclei were counterstained with TOPRO-3.

### Chemokine ELISA

Supernatants and cell lysates from triplicate wells of confluent primary HDLECs (either cultured in six-well dishes or on Transwell inserts (0.4 µm pore size; BD Biosciences), were assayed for CX3CL1, CCL2 and CCL5 using commercial antigen capture ELISA kits (DuoSet; R&D Systems), in accordance with the manufacturer's instructions. Supernatants were either applied directly to the ELISA or concentrated using Amicon Ultra 0.5 ml centrifugation tubes, 5 kDa cut-off (Millipore). Adherent cells were lysed in lysis buffer [50 mM Tris-HCl, pH 7.4, 100 mM NaCl, 1% NP-40 (v/v), 1 mM EDTA and protease inhibitor cocktail (Roche)] and then applied to the ELISA.

### Western blotting

MDDCs were lysed in NuPAGE LDS sample buffer (Invitrogen) and resolved on Bis-Tris 4–12% polyacrylamide SDS gels (NuPAGE; Invitrogen) with MES buffer, alongside SeeBlue standards (Invitrogen). Protein was transferred to Immobilon membranes (Millipore) and incubated with mouse anti-β-actin and rabbit anti-CX3CR1 antibodies, 0.2 µg/ml (AMS Biotechnology) overnight, followed by goat anti-mouse IRDye700 and goat anti-rabbit IRDye800 (Odyssey) in Odyssey blocking buffer. Blots were visualised using the Li-Cor Biosciences Odyssey imaging system.

### RT-PCR

Total cellular RNA was isolated (RNeasy, Qiagen) from HDLECs cultured for 24 hours in EGM-2 MV medium, either alone or supplemented with 2 ng/ml TNF-α. First-strand cDNA synthesis was carried out by Oligo dT priming (Invitrogen) using AMV reverse transcriptase (New England Biolabs), following the manufacturer's instructions. CX3CL1 transcripts were amplified using the primer pair CX3CL1Fwd2 (5-ATGGCTCCGATATCTCTGTCGT-3) and CX3CL1Rev2 (5-AAAAGCTCCGTGCCCACA-3), and β-actin with the primer pair ActinFwd (5-AGGCATCCTCACCCTGAAGTAC-3) and ActinRev (5-TTGCCAATGGTGATGACCTGGC-3). Products were resolved on 1.8% agarose-Tris-Borate-EDTA gels, alongside 100 bp DNA ladders (New England Biolabs).

### Flow cytometry

MDDCs, BMDCs and HDLECs were fixed in PFA-PBS, washed in PBS with 10% (v/v) FCS, then incubated with appropriate primary antibodies for 30 minutes at 4°C then washed in PBS with 5% (v/v) FCS, followed by incubation with goat antibodies against rat IgG conjugated to Alexa Fluor 488. For analysis of surface expression of CX3CL1 in HDLECs, cells were cultured for 24 hours in either EGM-2MV medium alone or with human TNF-α and/or Ilomastat, then lifted with Accutase in the presence of Ilomastat, to prevent subsequent shedding of CX3CL1. Cells were fixed in PFA-PBS and stained as for DCs. Measurement of MDDC β2 integrin activation was carried out as described previously (Johnson and Jackson, 2010). Cells were analysed by flow cytometry (CyAn, Dako Cytomation) using Flow Jo software.

### *In vitro* lymphatic endothelial transmigration assays

Primary HDLECs and MDLECs were seeded onto the underside (unless otherwise stated) of gelatin-coated FluoroBlok cell culture inserts (3 µm pore size; BD Biosciences), cultured until confluent and transmigration assays were performed as detailed previously (Johnson et al., 2006; Johnson and Jackson, 2010). Where indicated, cells were stimulated with TNF-α for 24 hours before use and CX3CL1 was added 2 hours before addition of MDDCs. Rabbit anti-human CX3CL1 neutralising antibodies (50 µg/ml, AMS Biotechnology) and goat anti-CCL21 neutralising antibodies (50 µg/ml, R&D Systems) were applied 30 minutes before the addition of MDDCs and maintained throughout the course of the experiment. To each well, 0.5×10^6^ fluorescently labelled MDDCs were applied at the start of the assay and numbers of MDDCs transmigrating through the filter and monolayer into the lower chamber were recorded on a fluorescent plate reader (Synergy HT; Bio-Tek) at 37°C using KC4 software (Biotech). Fluorescence emission was calibrated against a standard curve, and transmigration was expressed as the number of MDDCs in the lower chamber.

To compare migration of BMDC from wild-type mice with those from *Cx3cr1^−/−^* mice, Cell-Tracker-Green-labelled MHC class II^+^ DCs from age-matched donors (described above) were applied to MDLEC monolayers in parallel wells and fluorescence emission was calibrated against standard curves for wild-type and *Cx3cr1^−/−^* cells.

### *In vivo* assays for CX3CL1-mediated DC trafficking in inflamed mouse skin

Male BALB/c and C57Bl/6 mice aged 8–10 weeks were sensitised and challenged by topical application of oxazolone (4-ethoxymethylene-2 phenyl-2-oxazoline-5-one; Sigma-Aldrich) as previously described (Johnson et al., 2006). For FITC painting experiments, oxazolone-sensitised BALB/c mice were intraperitoneally injected with 0.4 mg of either rabbit anti-CX3CL1 neutralising antibodies (AMS Biotechnology) or rabbit IgG (R&D Systems) then challenged with 0.8% w/v oxazolone and 1.5 mg/ml FITC in 95% ethanol (v/v in water) after 24 hours and sacrificed after a further 24 hours. The draining cervical lymph nodes were removed and tissue was disrupted, passed through a 70 µm cell strainer (BD Biosciences) and analysed by flow cytometry (CyAn, DAKO). For adoptive transfer experiments, C57Bl/6 male mice were sensitised to oxazolone, and 5 days later, both ears were challenged by topical application of 0.8% oxazolone and 10^6^ Q-tracker655 (Molecular Probes, Invitrogen)-labelled BMDCs from littermates and 10^6^ Q-tracker525-labelled BMDCs from age-matched *Cx3cr1^−/−^* mice (described above) were dermally injected. After a further 24 or 48 hour period, animals were sacrificed and lymph nodes processed as detailed above. For visualising DCs post-injection, BMDCs were pre-labelled with either Cell Tracker Red or Cell Tracker Green dyes (Invitrogen), mixed in equal numbers and 10^5^ cells were injected. Dermis was then stained in whole-mount for LYVE-1^+^ lymphatics and frozen sections prepared of lymph nodes.

### Statistical analyses

The Mann–Whitney U-test was used to compare data sets throughout this study. *P*<0.05 was considered significant. For transmigration assays, statistical analyses were performed on data from the 8 hour time point.

## Supplementary Material

Supplementary Material

## References

[b1] Banerji S. Ni J. Wang S-X. Clasper S. Su J. Tammi R. Jones M. Jackson D. G. (1999). LYVE-1, a new homologue of the CD44 glycoprotein, is a lymph-specific receptor for hyaluronan. J. Cell Biol. 144, 789–801 10.1083/jcb.144.4.789 10037799 PMC2132933

[b2] Bazan J. F. Bacon K. B. Hardiman G. Wang W. Soo K. Rossi D. Greaves D. R. Zlotnik A. Schall T. J. (1997). A new class of membrane-bound chemokine with a CX3C motif. Nature 385, 640–644 10.1038/385640a0 9024663

[b3] Bourd-Boittin K. Basset L. Bonnier D. L'helgoualc'h A. Samson M. Théret N. (2009). CX3CL1/fractalkine shedding by human hepatic stellate cells: contribution to chronic inflammation in the liver. J. Cell. Mol. Med. 13 8A, 1526–1535 10.1111/j.1582-4934.2009.00787.x 19432809 PMC3828864

[b4] Dransfield I. Cabañas C. Barrett J. Hogg N. (1992). Interaction of leukocyte integrins with ligand is necessary but not sufficient for function. J. Cell Biol. 116, 1527–1535 10.1083/jcb.116.6.1527 1541641 PMC2289386

[b5] Fong A. M. Robinson L. A. Steeber D. A. Tedder T. F. Yoshie O. Imai T. Patel D. D. (1998). Fractalkine and CX3CR1 mediate a novel mechanism of leukocyte capture, firm adhesion, and activation under physiologic flow. J. Exp. Med. 188, 1413–1419 10.1084/jem.188.8.1413 9782118 PMC2213407

[b6] Förster R. Schubel A. Breitfeld D. Kremmer E. Renner-Müller I. Wolf E. Lipp M. (1999). CCR7 coordinates the primary immune response by establishing functional microenvironments in secondary lymphoid organs. Cell 99, 23–33 10.1016/S0092-8674(00)80059-8 10520991

[b7] Garton K. J. Gough P. J. Blobel C. P. Murphy G. Greaves D. R. Dempsey P. J. Raines E. W. (2001). Tumor necrosis factor-alpha-converting enzyme (ADAM17) mediates the cleavage and shedding of fractalkine (CX3CL1). J. Biol. Chem. 276, 37993–38001 11495925 10.1074/jbc.M106434200

[b8] Garton K. J. Gough P. J. Raines E. W. (2006). Emerging roles for ectodomain shedding in the regulation of inflammatory responses. J. Leukoc. Biol. 79, 1105–1116 10.1189/jlb.0106038 16565325

[b9] Geissmann F. Manz M. G. Jung S. Sieweke M. H. Merad M. Ley K. (2010). Development of monocytes, macrophages, and dendritic cells. Science 327, 656–661 10.1126/science.1178331 20133564 PMC2887389

[b10] Goda S. Imai T. Yoshie O. Yoneda O. Inoue H. Nagano Y. Okazaki T. Imai H. Bloom E. T. Domae N. (2000). CX3C-chemokine, fractalkine-enhanced adhesion of THP-1 cells to endothelial cells through integrin-dependent and -independent mechanisms. J. Immunol. 164, 4313–4320 10754331 10.4049/jimmunol.164.8.4313

[b11] Gunn M. D. Kyuwa S. Tam C. Kakiuchi T. Matsuzawa A. Williams L. T. Nakano H. (1999). Mice lacking expression of secondary lymphoid organ chemokine have defects in lymphocyte homing and dendritic cell localization. J. Exp. Med. 189, 451–460 10.1084/jem.189.3.451 9927507 PMC2192914

[b12] Haskell C. A. Cleary M. D. Charo I. F. (1999). Molecular uncoupling of fractalkine-mediated cell adhesion and signal transduction. Rapid flow arrest of CX3CR1-expressing cells is independent of G-protein activation. J. Biol. Chem. 274, 10053–10058 10.1074/jbc.274.15.10053 10187784

[b13] Huang Y. W. Su P. Liu G. Y. Crow M. R. Chaukos D. Yan H. Robinson L. A. (2009). Constitutive endocytosis of the chemokine CX3CL1 prevents its degradation by cell surface metalloproteases. J. Biol. Chem. 284, 29644–29653 10.1074/jbc.M109.045682 19723636 PMC2785596

[b14] Hundhausen C. Misztela D. Berkhout T. A. Broadway N. Saftig P. Reiss K. Hartmann D. Fahrenholz F. Postina R. Matthews V. (2003). The disintegrin-like metalloproteinase ADAM10 is involved in constitutive cleavage of CX3CL1 (fractalkine) and regulates CX3CL1-mediated cell-cell adhesion. Blood 102, 1186–1195 10.1182/blood-2002-12-3775 12714508

[b15] Imai T. Hieshima K. Haskell C. Baba M. Nagira M. Nishimura M. Kakizaki M. Takagi S. Nomiyama H. Schall T. J. (1997). Identification and molecular characterization of fractalkine receptor CX3CR1, which mediates both leukocyte migration and adhesion. Cell 91, 521–530 10.1016/S0092-8674(00)80438-9 9390561

[b16] Johnson L. A. Jackson D. G. (2010). Inflammation-induced secretion of CCL21 in lymphatic endothelium is a key regulator of integrin-mediated dendritic cell transmigration. Int. Immunol. 22, 839–849 10.1093/intimm/dxq435 20739459

[b17] Johnson L. A. Clasper S. Holt A. P. Lalor P. F. Baban D. Jackson D. G. (2006). An inflammation-induced mechanism for leukocyte transmigration across lymphatic vessel endothelium. J. Exp. Med. 203, 2763–2777 10.1084/jem.20051759 17116732 PMC2118156

[b18] Johnson L. A. Prevo R. Clasper S. Jackson D. G. (2007). Inflammation-induced uptake and degradation of the lymphatic endothelial hyaluronan receptor LYVE-1. J. Biol. Chem. 282, 33671–33680 10.1074/jbc.M702889200 17884820

[b19] Jung S. Aliberti J. Graemmel P. Sunshine M. J. Kreutzberg G. W. Sher A. Littman D. R. (2000). Analysis of fractalkine receptor CX(3)CR1 function by targeted deletion and green fluorescent protein reporter gene insertion. Mol. Cell. Biol. 20, 4106–4114 10.1128/MCB.20.11.4106-4114.2000 10805752 PMC85780

[b20] Kabashima K. Shiraishi N. Sugita K. Mori T. Onoue A. Kobayashi M. Sakabe J. Yoshiki R. Tamamura H. Fujii N. (2007). CXCL12-CXCR4 engagement is required for migration of cutaneous dendritic cells. Am. J. Pathol. 171, 1249–1257 10.2353/ajpath.2007.070225 17823289 PMC1988874

[b21] Kriehuber E. Breiteneder-Geleff S. Groeger M. Soleiman A. Schoppmann S. F. Stingl G. Kerjaschki D. Maurer D. (2001). Isolation and characterization of dermal lymphatic and blood endothelial cells reveal stable and functionally specialized cell lineages. J. Exp. Med. 194, 797–808 10.1084/jem.194.6.797 11560995 PMC2195953

[b22] Lämmermann T. Bader B. L. Monkley S. J. Worbs T. Wedlich-Söldner R. Hirsch K. Keller M. Förster R. Critchley D. R. Fässler R. (2008). Rapid leukocyte migration by integrin-independent flowing and squeezing. Nature 453, 51–55 10.1038/nature06887 18451854

[b23] Liu G. Y. Kulasingam V. Alexander R. T. Touret N. Fong A. M. Patel D. D. Robinson L. A. (2005). Recycling of the membrane-anchored chemokine, CX3CL1. J. Biol. Chem. 280, 19858–19866 10.1074/jbc.M413073200 15774461

[b24] Martín-Fontecha A. Sebastiani S. Höpken U. E. Uguccioni M. Lipp M. Lanzavecchia A. Sallusto F. (2003). Regulation of dendritic cell migration to the draining lymph node: impact on T lymphocyte traffic and priming. J. Exp. Med. 198, 615–621 10.1084/jem.20030448 12925677 PMC2194169

[b25] Moatti D. Faure S. Fumeron F. Amara M-W. Seknadji P. McDermott D. H. Debré P. Aumont M. C. Murphy P. M. de Prost D. (2001). Polymorphism in the fractalkine receptor CX3CR1 as a genetic risk factor for coronary artery disease. Blood 97, 1925–1928 10.1182/blood.V97.7.1925 11264153

[b26] Muehlhoefer A. Saubermann L. J. Gu X. Luedtke-Heckenkamp K. Xavier R. Blumberg R. S. Podolsky D. K. MacDermott R. P. Reinecker H. C. (2000). Fractalkine is an epithelial and endothelial cell-derived chemoattractant for intraepithelial lymphocytes in the small intestinal mucosa. J. Immunol. 164, 3368–3376 10706732 10.4049/jimmunol.164.6.3368

[b27] Ohl L. Mohaupt M. Czeloth N. Hintzen G. Kiafard Z. Zwirner J. Blankenstein T. Henning G. Förster R. (2004). CCR7 governs skin dendritic cell migration under inflammatory and steady-state conditions. Immunity 21, 279–288 10.1016/j.immuni.2004.06.014 15308107

[b28] Palframan R. T. Jung S. Cheng G. Weninger W. Luo Y. Dorf M. Littman D. R. Rollins B. J. Zweerink H. Rot A. (2001). Inflammatory chemokine transport and presentation in HEV: a remote control mechanism for monocyte recruitment to lymph nodes in inflamed tissues. J. Exp. Med. 194, 1361–1374 10.1084/jem.194.9.1361 11696600 PMC2195988

[b29] Pan Y. Lloyd C. Zhou H. Dolich S. Deeds J. Gonzalo J. A. Vath J. Gosselin M. Ma J. Dussault B. (1997). Neurotactin, a membrane-anchored chemokine upregulated in brain inflammation. Nature 387, 611–617 10.1038/42491 9177350

[b30] Patel D. D. Koopmann W. Imai T. Whichard L. P. Yoshie O. Krangel M. S. (2001). Chemokines have diverse abilities to form solid phase gradients. Clin. Immunol. 99, 43–52 10.1006/clim.2000.4997 11286540

[b31] Prevo R. Banerji S. Ferguson D. J. P. Clasper S. Jackson D. G. (2001). Mouse LYVE-1 is an endocytic receptor for hyaluronan in lymphatic endothelium. J. Biol. Chem. 276, 19420–19430 10.1074/jbc.M011004200 11278811

[b32] Qu C. Edwards E. W. Tacke F. Angeli V. Llodrá J. Sanchez-Schmitz G. Garin A. Haque N. S. Peters W. van Rooijen N. (2004). Role of CCR8 and other chemokine pathways in the migration of monocyte-derived dendritic cells to lymph nodes. J. Exp. Med. 200, 1231–1241 10.1084/jem.20032152 15534368 PMC2211916

[b33] Rao R. M. Yang L. Garcia-Cardena G. Luscinskas F. W. (2007). Endothelial-dependent mechanisms of leukocyte recruitment to the vascular wall. Circ. Res. 101, 234–247 10.1161/CIRCRESAHA.107.151860b 17673684

[b34] Saeki H. Moore A. M. Brown M. J. Hwang S. T. (1999). Cutting edge: secondary lymphoid-tissue chemokine (SLC) and CC chemokine receptor 7 (CCR7) participate in the emigration pathway of mature dendritic cells from the skin to regional lymph nodes. J. Immunol. 162, 2472–2475 10072485

[b35] Scheinecker C. McHugh R. Shevach E. M. Germain R. N. (2002). Constitutive presentation of a natural tissue autoantigen exclusively by dendritic cells in the draining lymph node. J. Exp. Med. 196, 1079–1090 10.1084/jem.20020991 12391019 PMC2194046

[b36] Steinman R. M. Hawiger D. Nussenzweig M. C. (2003). Tolerogenic dendritic cells. Annu. Rev. Immunol. 21, 685–711 10.1146/annurev.immunol.21.120601.141040 12615891

[b37] Tacke F. Alvarez D. Kaplan T. J. Jakubzick C. Spanbroek R. Llodra J. Garin A. Liu J. Mack M. van Rooijen N. (2007). Monocyte subsets differentially employ CCR2, CCR5, and CX3CR1 to accumulate within atherosclerotic plaques. J. Clin. Invest. 117, 185–194 10.1172/JCI28549 17200718 PMC1716202

[b38] Tal O. Lim H. Y. Gurevich I. Milo I. Shipony Z. Ng L. G. Angeli V. Shakhar G. (2011). DC mobilization from the skin requires docking to immobilized CCL21 on lymphatic endothelium and intralymphatic crawling. J. Exp. Med. 208, 2141–2153 10.1084/jem.20102392 21930767 PMC3182054

[b39] Umehara H. Goda S. Imai T. Nagano Y. Minami Y. Tanaka Y. Okazaki T. Bloom E. T. Domae N. (2001). Fractalkine, a CX3C-chemokine, functions predominantly as an adhesion molecule in monocytic cell line THP-1. Immunol. Cell Biol. 79, 298–302 10.1046/j.1440-1711.2001.01004.x 11380684

[b40] Viemann D. Goebeler M. Schmid S. Klimmek K. Sorg C. Ludwig S. Roth J. (2004). Transcriptional profiling of IKK2/NF-kappa B- and p38 MAP kinase-dependent gene expression in TNF-alpha-stimulated primary human endothelial cells. Blood 103, 3365–3373 10.1182/blood-2003-09-3296 14715628

[b41] Weber M. Hauschild R. Schwarz J. Moussion C. de Vries I. Legler D. F. Luther S. A. Bollenbach T. Sixt M. (2013). Interstitial dendritic cell guidance by haptotactic chemokine gradients. Science 339, 328–332 10.1126/science.1228456 23329049

[b42] White G. E. Greaves D. R. (2012). Fractalkine: a survivor's guide: chemokines as antiapoptotic mediators. Arterioscler. Thromb. Vasc. Biol. 32, 589–594 10.1161/ATVBAHA.111.237412 22247260

[b43] Wolff B. Burns A. R. Middleton J. Rot A. (1998). Endothelial cell “memory” of inflammatory stimulation: human venular endothelial cells store interleukin 8 in Weibel-Palade bodies. J. Exp. Med. 188, 1757–1762 10.1084/jem.188.9.1757 9802987 PMC2212526

[b44] Yoshikawa M. Nakajima T. Matsumoto K. Okada N. Tsukidate T. Iida M. Otori N. Haruna S. Moriyama H. Imai T. (2004). TNF-alpha and IL-4 regulate expression of fractalkine (CX3CL1) as a membrane-anchored proadhesive protein and soluble chemotactic peptide on human fibroblasts. FEBS Lett. 561, 105–110 10.1016/S0014-5793(04)00132-2 15013759

