# Supplementary material for: The chemokine CX3CL1 promotes trafficking of dendritic cells through inflamed lymphatics
Source: J Cell Sci. 2013 Nov 15;126(22):5259–70. doi: 10.1242/jcs.135343 (PMC3828594; doi:10.1242/jcs.135343)
Supplement: Supplementary Material [file supp_126_22_5259__index.html]

The chemokine CX3CL1 promotes trafficking of dendritic cells through inflamed lymphatics — Supplementary Material 

# The chemokine CX3CL1 promotes trafficking of dendritic cells through inflamed lymphatics

## JCS135343 Supplementary Material

**Files in this Data Supplement:**

- **Supplementary Material PDF**
