# Supplementary material for: The chemokine CX3CL1 promotes trafficking of dendritic cells through inflamed lymphatics
Source: J Cell Sci. 2013 Nov 15;126(22):5259–70. doi: 10.1242/jcs.135343 (PMC3828594; doi:10.1242/jcs.135343)
Supplement: Supplementary Material [file supp_126.22.5259_JCS135343.pdf]

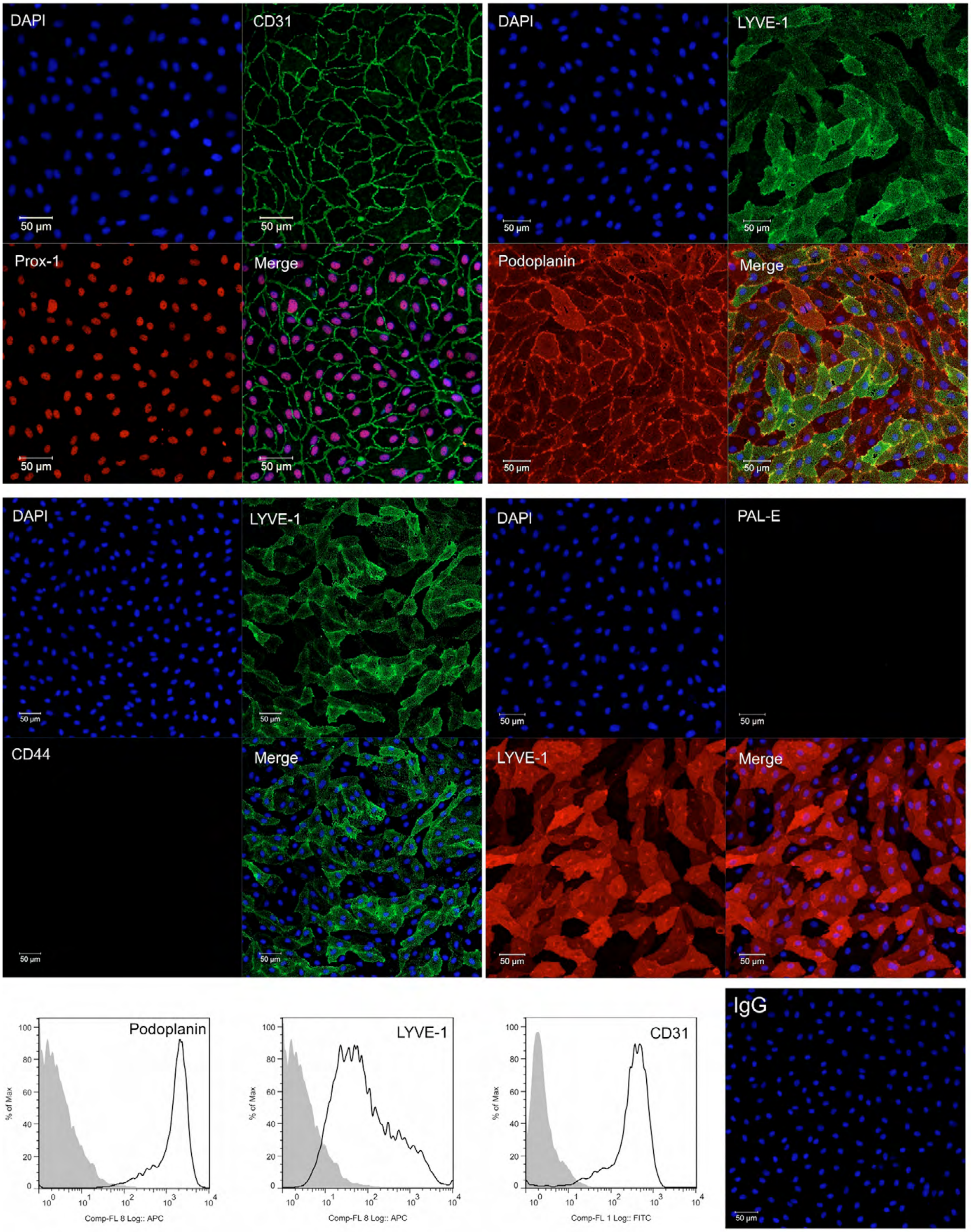


**Fig. S1. Phenotype of primary HDLEC.** Cells isolated from human dermis by LYVE-1 immunomagnetic bead selection are shown after immunofluorescence staining for lymphatic and blood vascular-specific markers. Confluent monolayers were found to express the pan-endothelial cell marker CD31, the lymphatic-endothelial specific transcription factor Prox-1 and the lymphatic markers podoplanin and LYVE-1. The heterogeneity in LYVE-1 expression reflects that observed in normal tissue lymphatics. HDLEC did not express the blood vascular endothelial cell marker PAL-E antigen, or CD44. Nuclei were counterstained with DAPI. Magnification: 100×. FACS histograms of HDLEC stained for podoplanin, LYVE-1 and CD31 (black) show that a pure population of HDLEC was obtained. Isotype controls are shown in grey.

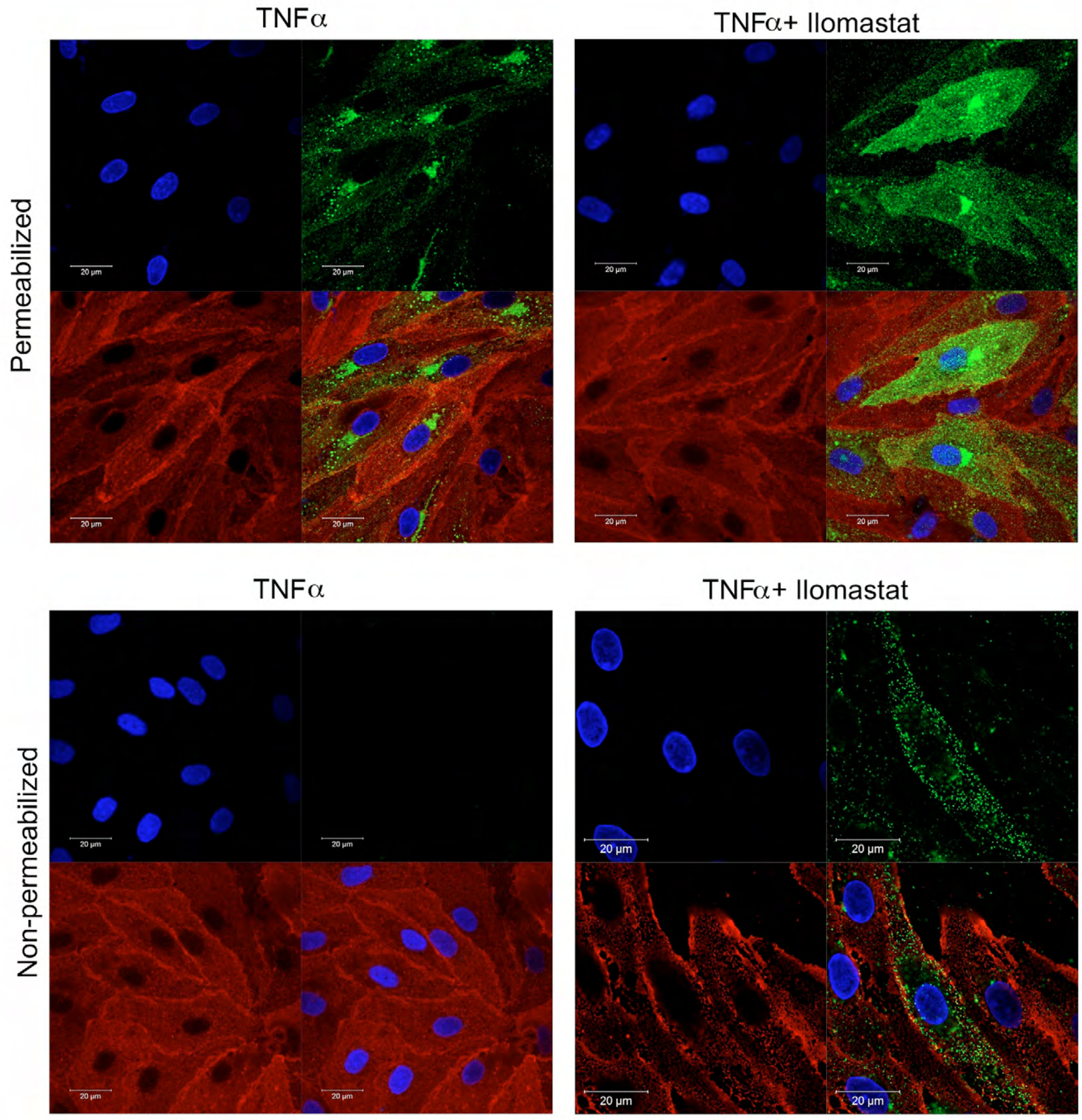


**Fig. S2. Expression of CX3CL1 in TNFα-stimulated HDLEC.** Split panels of the merged images shown in Fig. 2 are shown, following dual immunofluorescence staining of 24 h TNF-α-stimulated HDLEC for CX3CL1 (green) and podoplanin (red) in saponin-permeabilized and non-permeabilized HDLEC monolayers respectively. Nuclei were counterstained with DAPI, magnification: 6300×.

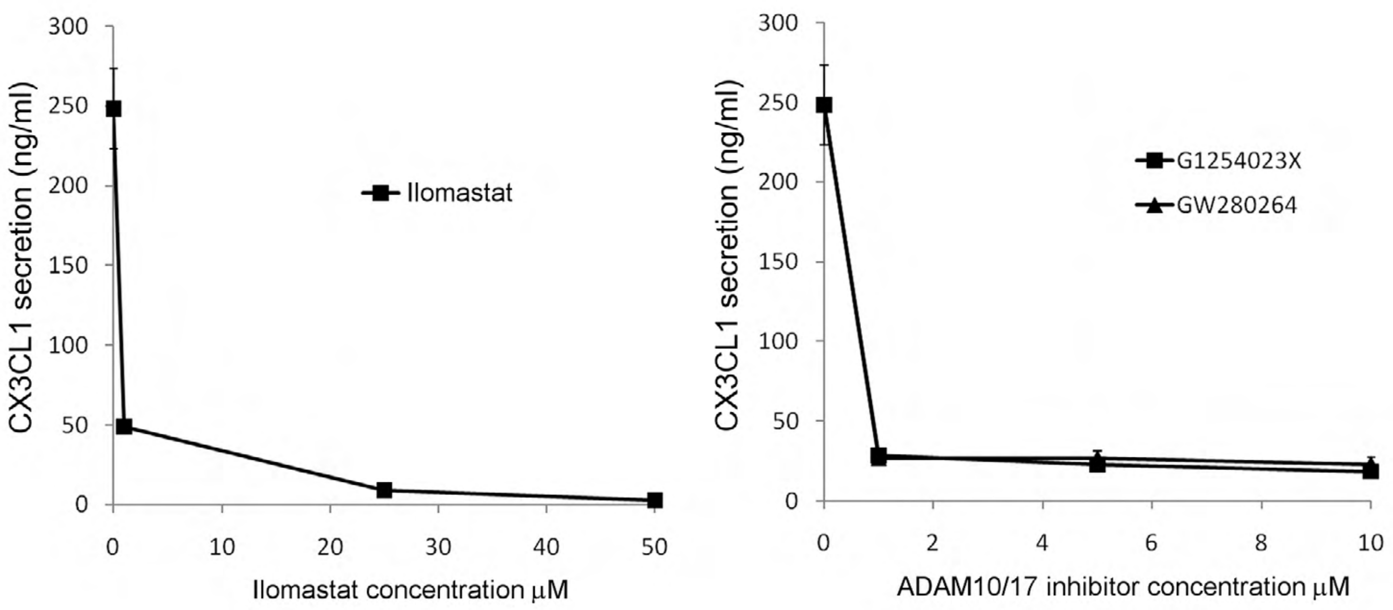


**Fig. S3. Effects of metalloproteinase inhibitors on CX3CL1 shedding from activated HDLEC.** HDLEC monolayers were stimulated with TNF-α in the presence of varying concentrations of either Ilomastat or the ADAM10/17 inhibitors GI254023X or GW280264X. Shedding of CX3CL1 into the supernatant was measured by ELISA, *n*=3.

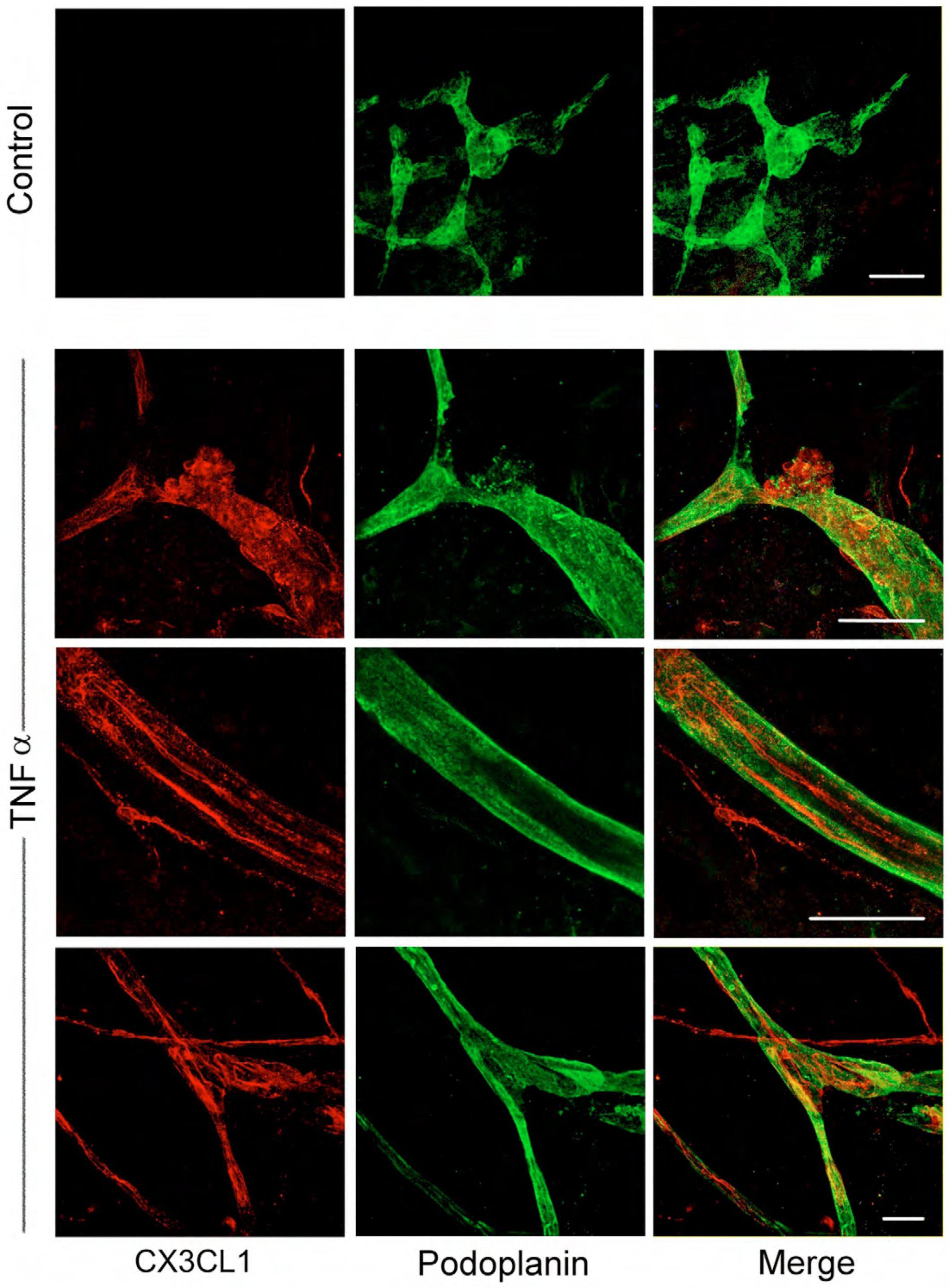


**Fig. S4. Expression of CX3CL1 in lymphatic endothelium *ex vivo*.** Whole-mount staining was performed on freshly resected (control) mouse ears and following TNF-α stimulation. Tissue was stained for podoplanin (green) and CX3CL1 (red). Representative images from four mice are shown (bars, top panel=100 μm, lower panels=50 μm).

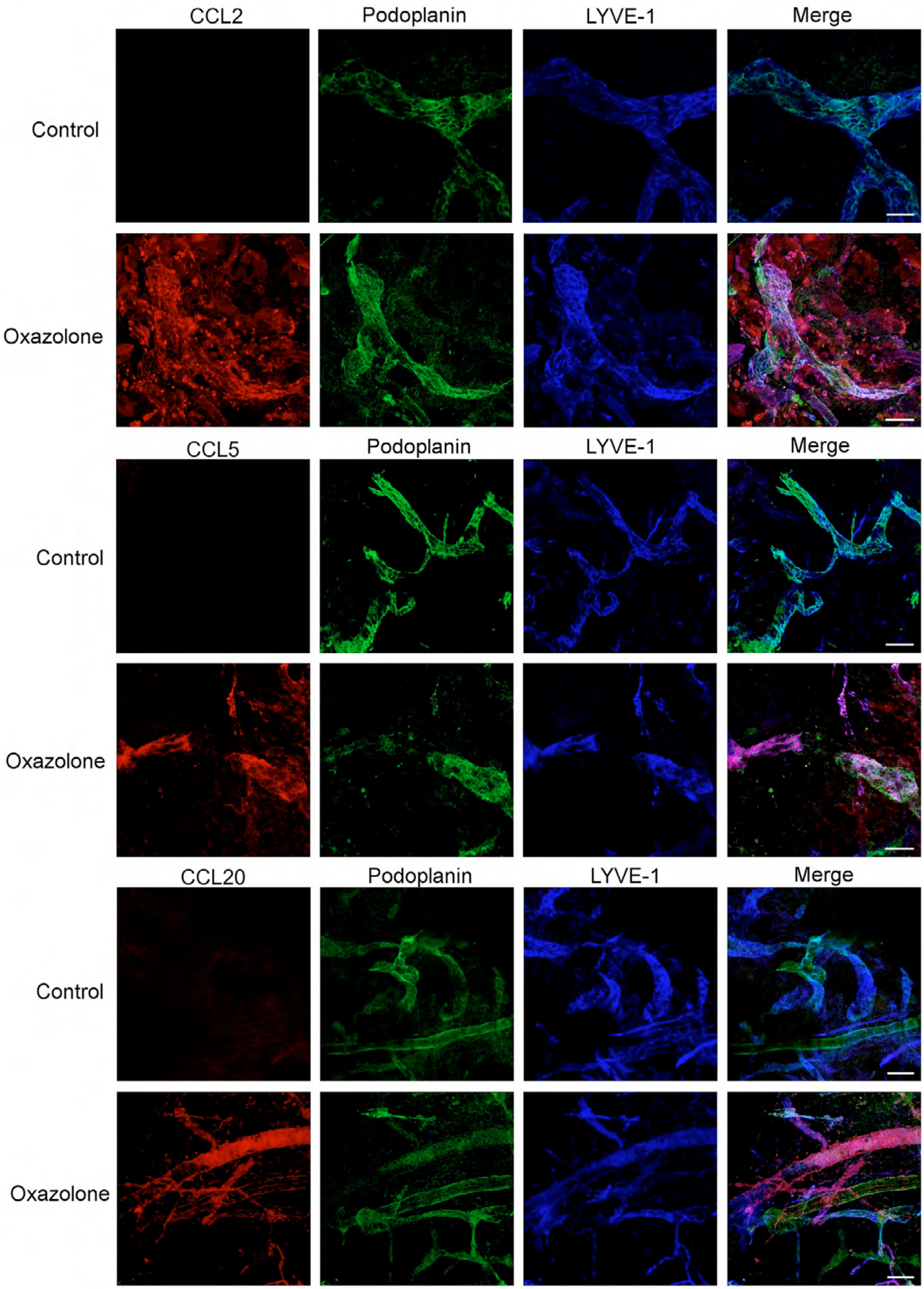


**Fig. S5. Induction of CX3CL1 and inflammatory CC chemokines in inflamed mouse dermal lymphatics *in vivo*.** Whole-mount staining for podoplanin (green), LYVE-1 (blue) and either CCL2, CCL5 or CCL20 as indicated (red) in ipsilateral inflamed ear dermis from mice subjected to topical oxazolone induced skin hypersensitivity, compared with the non-inflamed contralateral ear. Representative images from four mice are shown, (bar=100 µm).

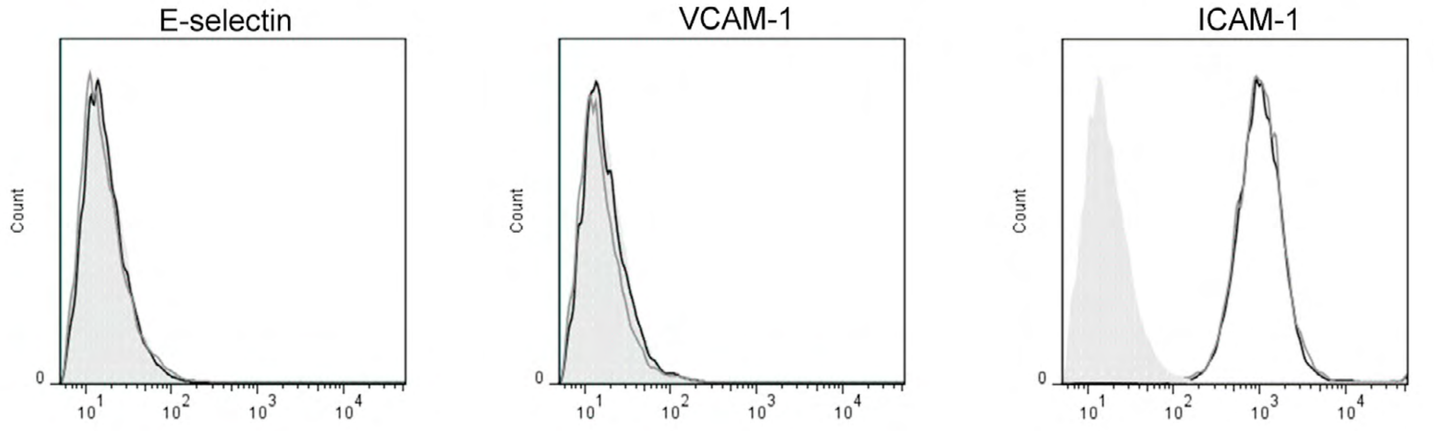


**Fig. S6. Exposure of HDLEC to exogenous CX3CL1 does not affect expression of CAMs.** Flow cytometry to measure expression of E-selectin, VCAM-1 and ICAM-1 was carried out on HDLEC following incubation for 4 h in either the presence (grey line) or absence (black line) of recombinant human CX3CL1. Irrelevant isotype-matched controls are shown in pale grey, one representative histogram of three shown in each case.

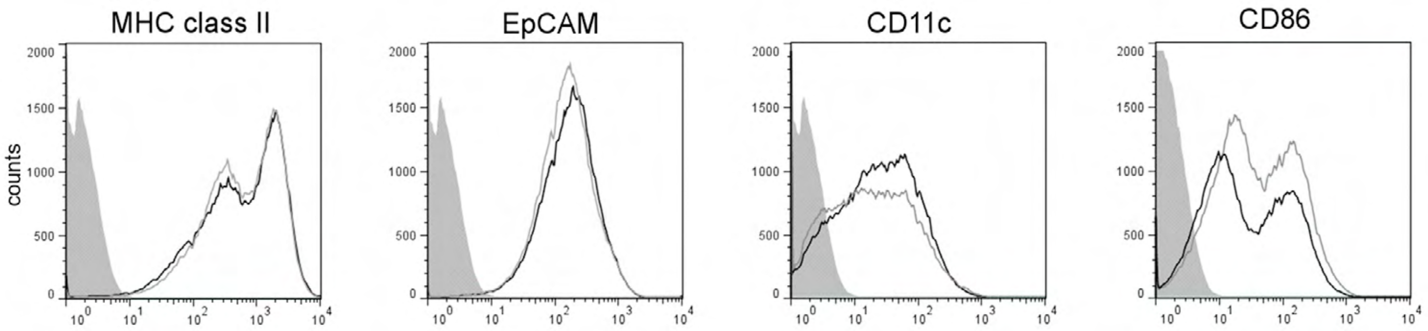


**Fig. S7. Phenotype of BMDC from wild-type and CX3CR1$^{-/-}$ mice.** Flow cytometry to show expression of cell surface markers on MHC class II$^{+}$ BMDC from wild-type (grey lines) and CX3CR1$^{-/-}$ mice (black lines), with isotype-matched controls depicted as filled grey histograms.

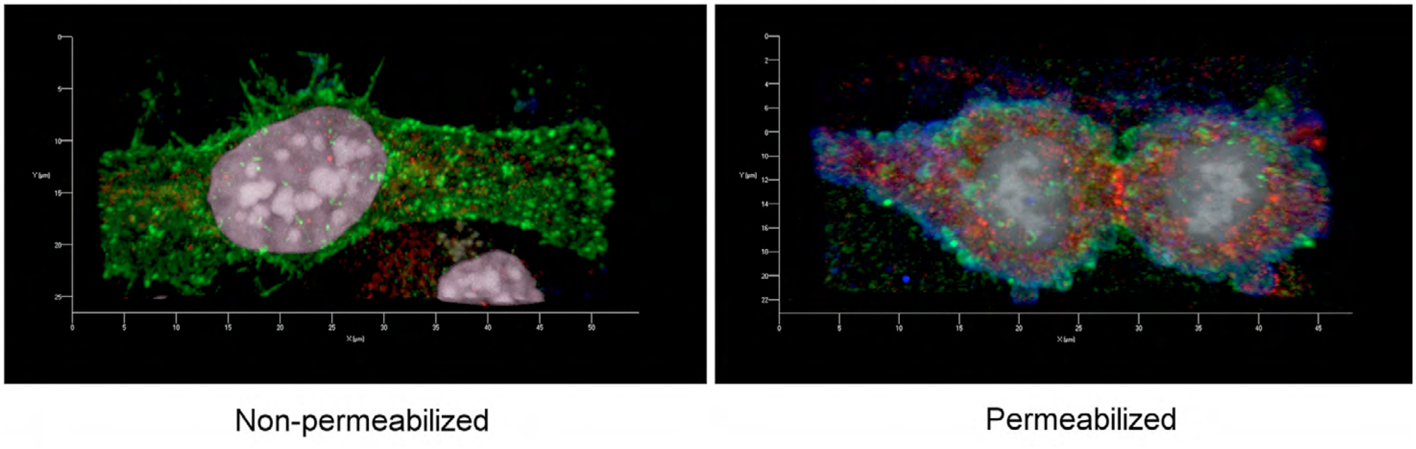


**Fig. S8. Expression of CX3CL1 in TNFα-stimulated MDLEC.** Staining for CX3CL1 (red), podoplanin (green) and LYVE-1 (blue) in control, non-permeabilized and saponin-permeabilized cells. Magnification: 630×.
